# Supplementary material for: Deciphering the molecular mechanisms of FET fusion oncoprotein–DNA hollow co-condensates
Source: Nat Commun. 2025 Nov 7;16:9823. doi: 10.1038/s41467-025-65069-4 (PMC12594852; doi:10.1038/s41467-025-65069-4)
Supplement: Supplementary file 1 — Supplementary Information [file 41467_2025_65069_MOESM1_ESM.pdf]

Supplementary Information

Deciphering the molecular mechanisms of FET fusion oncoprotein–DNA hollow co-condensates

Linyu Zuo[1, †], Qirui Guo[1, †], Cheng Li[1, †], Kecheng Zhang[2], Yancao Chen[1], Baiyi Jiang[1], Zhixing Chen[2,3], Yufei Xia[4, *], Long Qian[1, *], Lei Zhang[1, 5 *], and Zhi Qi[1, 6, *]

[†]Equal contribution

[1]Center for Quantitative Biology, Academy for Advanced Interdisciplinary Studies, Peking University, Beijing 100871, China

[2]Peking-Tsinghua Center for Life Sciences, Academy for Advanced Interdisciplinary Studies, Peking University, Beijing 100871, China

[3]College of Future Technology, Institute of Molecular Medicine, National Biomedical Imaging Center, Beijing Key Laboratory of Cardiometabolic Molecular Medicine, Peking University, Beijing 100871, China

[4]Key Laboratory of Biopharmaceutical Preparation and Delivery, Chinese Academy of Sciences, Beijing, 10090, China

[5]Beijing International Center for Mathematical Research, Center for Machine Learning Research, Peking University, Beijing 100871, China

[6]School of Physics, Peking University, Beijing 100871, China

*To whom correspondence should be addressed. Email: zhiqi7@pku.edu.cn; zhangl@math.pku.edu.cn; long.qian@pku.edu.cn; yfxia@ipe.ac.cn

Table of contents

1. Supplementary Methods

1.1 A mathematical model for hollow co-condensate formation

1.2 Imaging analysis

2. Protein sequences

3. Oligo Preparation

4. Supplementary Figures

5. Uncropped scans of all gels in Supplementary Figures

1. Supplementary Methods

1.1 A mathematical model for hollow co-condensate formation

1.1.1 Introduction

Since the discovery of phase separation induced biomolecular condensate formation, numerous numerical models have been developed to examine the dynamics of these macro-molecular structures. Depending on the level of detail they capture, these models can be broadly classified into microscopic or macroscopic categories. Microscopic models use molecular dynamics simulation techniques or their coarse-grained variations to simulate or approximate the potential structure of these self-assembled condensates [1-4]. They can capture high-resolution structural information about the component proteins and their interactions. For example, an atomistic simulation of an RNA-NDDX4 system was conducted, providing detailed molecular mechanisms involved in biomolecular condensate [5]. Similarly, this type of method was also used simulations to highlight the importance of nonspecific hydrophobic contacts in self-assembly [6]. However, microscopic models cannot escape the curse of dimensionality due to the complexity of modeling and sampling procedures, and their capabilities are largely constrained by computational resources.

To overcome these issues, several coarse-grained models have been employed to investigate system with higher complexity. For instance, Banerjee and co-workers [2] introduced a coarse-grained molecular dynamics simulation of an RNA-ribonucleoprotein (RNP) system to describe the process of hollow co-condensate formation. In their model, the side chains of each amino acid residue are treated as a single bead to reduce computational demands. For more complex proteins such as FUS, Wei and coworkers provided insights into the roles of its LCD domain in phase separation using a 100-ns coarse-grained molecular dynamics simulation of FUS and several of its derivatives [4]. Despite these advancements, coarse-grained models can only extend to systems on the scale of a few nanometers over a time span of a few nanoseconds. This scale is insufficient to cover the hollow formation procedures at the minute or millimeter scale, thereby limiting the applicability of microscopic models for simulating FUS systems.

Compared to microscopic models, macroscopic models operate at a coarser scale. Instead of detailing each molecule, macroscopic models typically use an energy functional that incorporates the inherent characteristics of, and the interactions between, the components within the system. For example, the Flory-Huggins theory-based model was used to comprehensively describe phase separation in multi-component mixtures [7,8]. The phase field model has also been introduced to represent phase-separated condensates using order parameters. These parameters denote the volume fractions of each component, thus describing the interactions between them. In this context, a Cahn-Hilliard phase-field model was used to explore RNA-protein interactions that form biomolecular condensate [3]. However, these continuum models primarily focus on interactions between components and largely overlook the internal

properties of the molecules. Therefore, a meso-scale simulation method that can capture the formation of condensates is highly desirable.

In this study, we propose a phase field model that incorporates molecular information and component interactions to account for FUS-associated hollow co-condensates. Specifically, we introduce two order parameters that represent macro-scale and micro-scale phase separation of the protein-dsDNA complex from solvents, and among themselves. We employ an energy functional based on the Ohta-Kawasaki model, integrating both order parameters to depict the formation and structure of the condensates. With respect to DNA, we introduce a particular order parameter to reflect on the existence of DNA molecules within DNA-protein complex. The minimization of the energy functional naturally leads to an equilibrium structure. Through computational simulation, our model successfully replicates the hollow co-condensates observed in experiments. Furthermore, the molecule-informed parameters within our model demonstrate strong correlation with actual conditions across several control experiments. Here we will briefly report the development of the model and numerical simulation results.

1.1.2 Model development

Suppose that there are at least two components in the system: FUS-ERG-dsDNA complex C and solvent molecule S. Based on the observed amphiphilic property of DNA-bound FUS-ERG protein in this study. We assume that the DNA-protein complex C composed of hydrophilic parts C_{h} and hydrophobic parts C_b. Motivated by Ohta-Kawasaki 's di-block copolymer model [9,10] and Han's model [11] upon the amphiphilic lipid bilayers, we adopt similar energy functional in Landau-Ginzburg's manner to describe protein backbones in the system. In particular, we assume that the state of

protein-DNA-solvent system could be described by two independent order parameters ϕ, η.

$$\phi = \zeta_{C_h} - \zeta_{C_b}$$

$$\eta = \zeta_{C_h} + \zeta_{C_b} - \psi_C$$

Here $\zeta_{\cdot}$ denotes the local volume fractions of each component and ψ_C is the critical volume fraction for phase separation emergence. The parameter η could reflects on the overall DNA-protein complex concentration distribution i.e., the macroscopic phase separation between complex and solvent. Notably, the parameter ϕ denotes for the local difference between parts of the components, known as microscopic phase separation. Total free energy E_F involving these order parameters consists of two parts: short range energy E_S and long-range part E_L [9].

$$E_F\{\phi, \eta\} = E_S\{\phi, \eta\} + E_L\{\phi, \eta\}$$

As for short range one, we adopt Ginzburg-Landau form by

$$E_S\{\phi, \eta\} = \int \left[\frac{c_\eta}{2}|\nabla\eta|^2 + \frac{c_\phi}{2}|\nabla\phi|^2 + W(\phi, \eta)\right] dr$$

where c_1, c_2 re model parameters governing the interface thickness of each component and $W(\phi, \eta)$ is a potential function. Ito adopts the Flory-Huggins approximation in this model [10] and assume $W(\phi, \eta)$ takes form of

$$W(\phi, \eta) = \frac{(\eta^2 - 1)^2}{4} + \frac{(\phi^2 - 1)^2}{4} + I(\eta, \phi)$$

$$I(\phi, \eta) = b_1\eta\phi - \frac{b_2}{2}\eta\phi^2 - \frac{b_3}{2}\eta^2\phi + \frac{b_4}{2}\eta^2\phi^2$$

where $I(\phi, \eta)$ is a phenomenological energy term denoting the interactions between these two components. Specifically, the first term $\eta \cdot \phi$ denotes molecular interaction and its scale b_1 is given by

$$b_1 = \frac{1}{4}\left(u_{C_hC_h} - u_{C_bC_b}\right) - \frac{1}{2}\left(u_{C_hS} - u_{C_bS}\right)$$

where $u_{\cdot,\cdot}$ represents interaction strength between components. Other terms in the interaction function I mainly arise from the confrontational entropy and indicate the coupling between phase separations of different scales. In the original Ohta-Kawasaki model, these parameters are selected based on the copolymer's characteristics such as polymerization indices or block ratio. Here we will treat them as phenomenological ones instead. These parameters are selected such that overall potential function W has three individual minimizers, which correspond to solvent, hydrophilic and hydrophobic parts of the complex. Besides local interactions within complex, connectivity between parts further leads to the long-range energy term with the form of:

$$E_L\{\phi,\eta\} = \iint G(x,x')[\alpha\delta\phi(x)\delta\phi(x')$$

$$+\beta\delta\eta(x)\delta\phi(x') + \gamma\delta\eta(x)\delta\eta(x')\,]\,dxdx'$$

$$\delta\eta = \eta - \bar{\eta}$$

$$\delta\phi = \phi - \bar{\phi}$$

where $G(r,r')$ is the Green's function defined as $-\nabla^2 G(x,x') = \delta(x-x')$. $\bar{\eta},\bar{\phi}$ represent the spatial mean of order parameter η,ϕ respectively. Coefficients α,β,γ describe the block information of copolymers and $\beta = \gamma = 0$ for diblock co-polymers in the original OK model. Here we take α as a positive coefficient to represent linkage between proteins' hydrophilic and hydrophobic parts and simply neglect β,γ by setting them to 0.

Given the free energy functional, stable structures of the system cloud be retained by finding the minimizers. Instead of analytical solving the optimal η, ϕ, we build the dynamics of them in the Cahn-Hilliard's scheme given transport coefficients L_η, L_ϕ as

$$\frac{\partial \eta}{\partial t} = L_\eta \nabla^2 \frac{\delta E_F}{\delta \eta}$$

$$\frac{\partial \phi}{\partial t} = L_\phi \nabla^2 \frac{\delta E_F}{\delta \phi}$$

Based on the dynamics of the protein-DNA complex, we also tried to emphasize dsDNA alongside the simulation. Given that protein of interests in this work FUS-ERG contains DNA-binding domains with high affinity. We assume that most of the dsDNA molecules are absorbed into protein condensates. For dsDNA bound to proteins and distribution order parameter χ, we build its dynamics by

$$\left.\frac{\partial \chi}{\partial t}\right|_{In\,protein} = D_\chi \nabla^2 \chi + a_\chi - d_\chi \cdot \chi$$

Apparently, changes in the volume fraction of dsDNA bound to proteins are affected by diffusion, absorption and decay effect with coefficient D, a, d respectively. These coefficients reflect on the affinity of DNA molecules to proteins and the ability to exist within the protein-DNA complex. Inspired by the previous reference [12] we introduce another order parameter ν based on values of η, ϕ to track the region of the protein. Coupling ν and dynamics above, the overall dynamics of dsDNA could be rearranged as

$$\frac{\partial(\nu \cdot \chi)}{\partial t} = D_\chi \nabla \cdot (\nu \cdot \nabla \chi) + \nu(a_\chi - d_\chi \cdot \chi)$$

Based on the affinity of dsDNA to parts of protein, ν could be arbitrarily selected. Here based on the potential affinity of DBD, we simply choose $\nu = \max(-\phi, 0)$.

Combining dynamics of complex and dsDNA, we model the dynamics of condensation of FUS-ERG-DNA system in terms of η, ϕ, χ.

$$\frac{\partial \eta}{\partial t} = L_\eta \nabla^2 \frac{\delta E_F}{\delta \eta}$$

$$\frac{\partial \phi}{\partial t} = L_\phi \nabla^2 \frac{\delta E_F}{\delta \phi}$$

$$\frac{\partial (\nu \cdot \chi)}{\partial t} = D_\chi \nabla \cdot (\nu \cdot \nabla \chi) + \nu (a_\chi - d_\chi \cdot \chi)$$

1.1.3 Numerical simulation

Without loss of generality, we carry out the numerical simulation of the non-linear partial differential equations system on a 2D grid with 128 × 128 uniform grid points and choose periodic boundary conditions. We also apply semi-implicit Fourier-spectral method to ensure an acceptable step length [13]. Given weak correlation between dynamics of protein and that of dsDNA. We first try to discretized the first two equations involving η, ϕ.

$$\frac{\eta^{(t+1)} - \eta^{(t)}}{\Delta t} = \nabla^2 N_\eta\left(\eta^{(t)}\right) + L_\eta\left(\eta^{(t+1)}\right)$$

$$\frac{\phi^{(t+1)} - \phi^{(t)}}{\Delta t} = \nabla^2 N_\phi\left(\phi^{(t)}\right) + L_\phi\left(\phi^{(t+1)}\right)$$

Where N, L denotes non-linear term and linear term in $\nabla^2 \delta E_F / \delta$. In particular, we have

$$L_\eta = \nabla^2\left(b_1 \phi - \eta - c_\eta \nabla^2 \eta\right) - \beta \delta \phi - \gamma \delta \eta$$

$$N_\eta = \eta^3 - b_2 \phi^2 / 2 - b_3 \eta \phi + b_4 \eta \phi^2$$

$$L_\phi = \nabla^2\left(b_1 \eta - \phi - c_\phi \nabla^2 \phi\right) - \alpha \delta \phi - \beta \delta \phi$$

$$N_\phi = \phi^3 - b_2 \phi \eta - b_3 \eta^2 / 2 + b_4 \eta^2 \phi$$

Fourier-spectral method solves the iteration in frequency domain given wave number vector $\mathbf{k} = (k_x, k_y)$ and $k = |\mathbf{k}|$ as the magnitude.

$$\eta(r,t) = \frac{1}{MN} \sum_{k_x=-N/2}^{N/2} \sum_{k_y=-M/2}^{M/2} \hat{\eta}(k,t) e^{ik\cdot r}$$

$$\phi(r,t) = \frac{1}{MN} \sum_{k_x=-N/2}^{N/2} \sum_{k_y=-M/2}^{M/2} \hat{\phi}(k,t) e^{ik\cdot r}$$

The iteration in frequency domain goes like

$$\left(1 - k^2 \Delta t + c_\eta k^4 \Delta t\right)\widehat{\eta^{(t+1)}} + \gamma \Delta t \delta \widehat{\eta^{(t+1)}} + b_1 k^2 \Delta t \widehat{\phi^{(t+1)}} + \beta \Delta t \delta \widehat{\phi^{(t+1)}}$$

$$= \widehat{\eta^{(t)}} - k^2 \Delta t \widehat{N_\eta(\eta^{(t)})}$$

$$b_1 k^2 \Delta t \widehat{\eta^{(t+1)}} + \beta \Delta t \delta \widehat{\eta^{(t+1)}} + \left(1 - k^2 \Delta t + c_\phi k^4 \Delta t\right)\widehat{\phi^{(t+1)}} + \alpha \Delta t \delta \widehat{\phi^{(t+1)}}$$

$$= \widehat{\phi^{(t)}} - k^2 \Delta t \widehat{N_\phi(\phi^{(t)})}$$

Rearrange of the system gives the linear system as

$$\begin{pmatrix} 1 + \left(k^2\left(c_\eta k^2 - 1\right) + \gamma\right)\Delta t & (b_1 k^2 + \beta)\Delta t \\ (b_1 k^2 + \beta)\Delta t & 1 + \left(k^2\left(c_\phi k^2 - 1\right) + \alpha\right)\Delta t \end{pmatrix} \begin{pmatrix} \hat{\eta}^{(t+1)} \\ \hat{\phi}^{(t+1)} \end{pmatrix}$$

$$= \begin{pmatrix} \widehat{\eta^{(t)}} - k^2 \Delta t \widehat{N_\eta(\eta^{(t)})} \\ \widehat{\phi^{(t)}} - k^2 \Delta t \widehat{N_\phi(\phi^{(t)})} \end{pmatrix}$$

Similarly, we conduct discretization on DNA dynamics. Before that we first expand the $\nabla \cdot$ part as

$$\frac{\partial(\nu \cdot \chi)}{\partial t} = D_\chi(\nu \cdot \nabla^2 \chi + \nabla \nu \cdot \nabla \chi) + \nu\left(a_\chi - d_\chi \chi\right)$$

During each iteration, we have such split form

$$\frac{\chi^{(t+1)} - \chi^{(t)}}{\Delta t} = \underbrace{D_\chi \nabla^2 \chi^{(t+1)} + \left(a_\chi - d_\chi \chi^{(t+1)}\right)}_{Linear} + \underbrace{\frac{D_\chi}{\nu} \nabla \nu \cdot \nabla \chi^{(t)}}_{Non\ Linear}$$

Fourier transform of the equation above gives

$$\widehat{\chi^{(t+1)}} - \widehat{\chi^{(t)}} = -D_\chi k^2 \Delta t \widehat{\chi^{(t+1)}} + \widehat{a_\chi} - d_\chi \Delta t \widehat{\chi^{(t+1)}} + \frac{D_\chi \Delta t}{\nu} \widehat{(\nabla \nu)} * \widehat{\nabla \chi^{(t)}}$$

where $*$ denotes convolution operation in frequency domain. To compute such term, we turn to the convolution theorem by

$$(1 + D_\chi k^2 \Delta t + d_\chi \Delta t) \widehat{\chi^{(t+1)}} = \widehat{\chi^{(t)}} + \widehat{a_\chi} + D_\chi \Delta t C\left(\widehat{\chi^{(t)}}\right)$$

$$C\left(\widehat{\chi^{(t)}}\right) = \mathcal{F}\left(\mathcal{F}^{-1}(ik_x \hat{\nu})/\nu \cdot \mathcal{F}^{-1}\left(ik_x \widehat{\chi^{(t)}}\right) + \mathcal{F}^{-1}(ik_x \hat{\nu})/\nu \cdot \mathcal{F}^{-1}\left(ik_x \widehat{\chi^{(t)}}\right)\right)$$

where $\mathcal{F}, \mathcal{F}^{-1}$ are Fourier and inverse Fourier operator respectively. Solving such equation gives the updating scheme of χ

$$\widehat{\chi^{(t+1)}} = \left(\widehat{\chi^{(t)}} + \widehat{a_\chi} + D_\chi \Delta t C\left(\widehat{\chi^{(t)}}\right)\right) / (1 + D_\chi k^2 \Delta t + d_\chi \Delta t)$$

$$C\left(\widehat{\chi^{(t)}}\right) = \mathcal{F}\left(\mathcal{F}^{-1}(ik_x \hat{\nu})/\nu \cdot \mathcal{F}^{-1}\left(ik_x \widehat{\chi^{(t)}}\right) + \mathcal{F}^{-1}(ik_x \hat{\nu})/\nu \cdot \mathcal{F}^{-1}\left(ik_x \widehat{\chi^{(t)}}\right)\right)$$

We also note all model coefficients in the following Table 1:

Table 1. Model coefficients of this mathematical model for hollow co-condensate formation.

Parameter	Meaning	Value
M, N	Grid Size	128
Δt	Simulation step size	0.2
c_η	Interface thickness	0.6
c_ϕ	Interface thickness	0.6
L_η, L_ϕ	Transport coefficient	1
ψ_C	Local critical protein concentration	0.85
b_1	Interaction strength between components	0.14

b_2	Coupling between condensates formation and protein concentration	0.4
b_3	Coupling between condensates formation and protein concentration	-0.425
b_4	Coupling between micro/macro-scope phase separation	0.85
α	Connections between parts of protein	0.008
β, γ	Copolymer coefficient (Neglected)	0
D_χ	Diffusion coefficient of dsDNA within protein context	0.3
a_χ	DNA absorption rate of protein	7×10^{-3}
d_χ	Erosion rate of dsDNA	5×10^{-4}

1.1.4 Simulation details

Initially, our focus is on the process of hollow co-condensate formation. As per experimental results, the FUS-ERG protein first undergoes a phase-separation process to form droplets. When dsDNA is introduced into the system, it first co-localizes within the protein droplet, and gradually, hollow co-condensates composed of DNA-protein complexes begin to emerge. It is also demonstrated that the composition of the external surface differs from that of the internal one: the external surface mirrors the structure of the initial FUS-ERG condensates, while the internal surface displays a higher concentration of RGG motifs. Based on these observations, we make the following assumptions: (i) both FUS-ERG and DNA-bound FUS-ERG proteins are amphiphilic molecules; (ii) the LCD FUS domain serves as the hydrophilic part, while the ERG motif exhibits hydrophobic characteristics in the FUS-ERG complex. Once combined with dsDNA molecules, the RGG motif in DNA-bound FUS-

ERG acts as hydrophilic segments, while the rest remains hydrophobic; (iii) during hollow co-condensate formation, the composition of the external surface remains unchanged.

We first constructed a protein droplet as the initial state, with a layer of hydrophilic domains of the protein enriched on the droplet surface, where dsDNA is attracted by the protein and distributed on the surface of the protein droplet. Before simulation, dsDNA molecules will first be transferred into the droplet through diffusion. Then we initiate the numerical simulation until equilibrium. During such process we constrain dsDNA dynamics within region $\nu = \max(0, -\phi)$. As demonstrated in Fig. 4a and d, starting from a droplet, an internal surface with higher microscopic phase separation ϕ gradually emerges. Concurrently, changes in macroscopic phase separation η, i.e., the overall distribution of protein-DNA complex, indicate the formation of hollow co-condensates. A strong correlation between dsDNA concentration χ and ϕ is also observable. In the early stages of condensate formation, the motion of dsDNA is accompanied by the dynamics of protein concentration, producing a cavity in the droplet. The dynamics of dsDNA further accounts for its asymmetric distribution on stabilized protein condensates, which align closely with the experimental results.

To investigate effects of protein-to-DNA molar ratio in hollow co-condensate formation, we further consider systems with different DNA/protein concentration by adjusting the protein and dsDNA concentration in the initial states as in Fig. 4a(i) respectively and perform simulation. We record the final structure of each case and plot the phase diagram in Fig. 4c. As indicated by the result, the hollow co-condensates prefer scenario with abundant dsDNA and protein concentration, which is consistent with the experimental observations Fig.1c(ii).

In this study, we extend our inquiry beyond the replication of experimental outcomes to engage in a discussion concerning the significance of model coefficients and their interplay with factors that influence the formation of condensates. Given the phenomenological nature of all parameters under consideration, our analysis will predominantly adopt a qualitative approach towards understanding the influence exerted by these coefficients, with a particular emphasis on b_1 and ψ_C.

As previously elucidated, the coefficient b_1 is potentially indicative of the interactions between compositions within the system. An elevated value of b_1 suggests an increased repulsive force between the hydrophobic regions of DNA-protein complex and the solvent, or conversely, higher affinity between the hydrophilic regions and the solvent. We then conduct simulation sharing other conditions yet under lower $b_1' = 0.04$ and observe that the stable structure turns out to be homogeneous condensates compared to hollow co-condensates under normal $b_1 = 0.14$ (Supplementary Fig. 9b). At lower b_1 values, the interactions between monomers are insufficiently robust to facilitate interface formation, leading to the disappearance of hollow co-condensates. These findings are corroborated by experimental observations in GFP-FUS-ERG(9RA) or GFP-FUS-DDIT3 mutants. In the case of DNA-bound FUS fusion proteins, mutations in the RGG motif result in a diminished volume of positive charges, thereby reducing the affinity of the hydrophilic component. Such mutants are predisposed to forming homogeneous condensates, rather than hollow co-condensates. The influence of b_1 on this process underscores the critical role of the RGG motif in the formation of hollow structures and aligns with previous hypotheses regarding the regulatory function of RGG.

We then cast sight to another important coefficient ψ_C. Such coefficient directly determines the order parameter η and controls “effective” phase separation volume at

specific protein concentration. Given the intrinsic coupling between η and ϕ, alongside the conservation principles inherent in Cahn-Hilliard dynamics, ψ_C plays a decisive role in dictating the equilibrium structure of the system. The interaction terms $\eta\phi^2$, $\eta^2\phi$ and $\eta^2\phi^2$ within the short-range energy function E_S suggest that the manifestation of microscopic phase separation is contingent upon a sufficient magnitude of η, which, in turn, is dependent on ψ_C. An increase in ψ_C results in a corresponding decrease in η for any given constant protein concentration, thereby impeding the formation of internal surfaces during the condensation process. As shown in Supplementary Fig. 9a, simulations conducted under higher $\psi_C = 0.95$ further indicates the formation of homogeneous condensates. In this context, we posit that ψ_C within our model correlates with the presence of “crosslinker” dsDNA molecules within protein condensates. Previous discussions have highlighted that dsDNA capable of inducing hollow formations possesses GGAA microsatellite sequences, which exhibit a high affinity with the DNA-binding domain in FUS-ERG proteins. The presence of multiple copies of such microsatellites on a single dsDNA molecule may facilitate the recruitment of multiple protein entities, a phenomenon akin to "cross-linking" observed in polymer materials. This cross-linking capability significantly reduces the local monomer concentration required for polymerization. Consequently, a higher propensity of dsDNA to aggregate proteins corresponds to a reduced concentration of proteins necessary to initiate self-assembly, i.e., a lower ψ_C. The inability to form hollow co-condensates with randomly sequenced dsDNA, which lacks cooperative interactions with proteins, further underscores the critical importance of ψ_C.

Besides the protein backbone, we extend our exploration to encompass the distribution of dsDNA within hollow co-condensates, with a particular focus on the interactions between the GGAA motif and proteins. By coupling the dsDNA dynamics

to hydrophobic ERG DBD of protein $\nu = \max(0, -\phi)$, we are able to predict the probable regions of dsDNA distribution. The simulation outcomes (Fig. 4a(ii)) under these conditions have successfully mirrored experimental observations, thus providing a robust validation of our model's predictive accuracy. Moreover, we delve into an additional scenario, as documented in the literature, which involves the presence of protein recognition motifs (PRM) and RNA within the condensates. In these systems, RNA first form tadpole-like amphiphilic deblock copolymer with the PRM protein and drive the formation of hollow co-condensates. This phenomenon can be conceptualized as a shift in the coupling region ν of nucleic acids from $\max(0, -\phi)$ to $\max(0, \phi + \eta)$. The simulations (Fig. 4e) conducted to replicate this scenario have not only depicted a symmetric distribution of components but have also aligned with the trends observed in RNA signal strength within STED microscopy data (Fig. 3g(ii)). This congruence further underscores the fidelity of our model in capturing the nuanced dynamics of molecular interactions within condensates.

We also conducted simulations involving multiple DNA species to illustrate the potential selectivity of DNA-protein condensates. Under such scenario we assume that all protein molecules form complexes with various DNA types, with minimal interaction between different DNA species. This allowed us to represent various DNA species using distinct parameters a, d, D as previously described. Without loss of generality, we mainly focused on two types of DNA: a high-affinity species (H) and a low-affinity species (L). The parameters for these are detailed in the accompanying table. In the first scenario, proteins are first mixed with high-affinity DNA (H), while low-affinity DNA (L) was placed around the complex, as shown in Supplementary Fig. 10a(i). We preserve the same model coefficients as in the main simulation of FUS-ERG-induced hollow condensates. As the simulation progressed, the hollow structure emerges, with

high-affinity DNA distributed throughout the condensates (Supplementary Fig. 10a(ii)). These observations highlight the potential impact of DNA selectivity on the final condensate structure and the corresponding DNA distribution. We also note all DNA-related coefficients in this multi-species model in the following Table 2:

Table 2. All DNA-related coefficients in this multi-species model.

Coefficients	For Higher-affinity DNA (H)	For Lower-affinity DNA (L)
a	1.5×10^{-4}	0
d	1×10^{-4}	1×10^{-4}
D	0.3	0.1

In summary, the model employed in this study has adeptly replicated the observed phenomena of hollow co-condensate formation, demonstrating its robustness and predictive accuracy. Through a detailed examination of model parameters, we have established a meaningful connection between phenomenological coefficients and the empirical factors observed in experiments. This analysis not only elucidates the underlying mechanisms of condensate formation but also opens avenues for designing experimental conditions conducive to the induction of hollow co-condensates. Furthermore, the congruence between our theoretical predictions and experimental observations regarding dsDNA distribution within condensates underscores the model's capacity to accurately capture the spatial organization of nucleic acids in these complex systems. The simulation of a system comprising multiple types of dsDNA has highlighted the potential of FUS-ERG condensates to selectively interact with, and possibly store, specific dsDNA sequences. These findings suggest a nuanced role of condensates in cellular processes, potentially

extending beyond mere phase separation to include functions such as molecular sorting and storage.

1.2 Imaging analysis

Analysis of STED data analysis consists of three main steps: Enhancement, Annotation and Measurements. The initial step focuses on enhancing the quality of the microscopy images to facilitate a clearer understanding of the structures being examined. This is achieved through a process that begins with the application of a discrete Fourier transformation $\hat{x} = \mathcal{F}(x)$ to the input image x, effectively translating it into the Fourier K-space. This transformation is pivotal as it allows for the assumption that white noise, which typically manifests as high-frequency components in the spatial domain, corresponds to constants in the K-space. To mitigate the impact of this noise, a low-pass filter is applied to eliminate all frequency components above a predetermined threshold, typically set at half the grid length. By zeroing out these high-frequency components, the process effectively removes the noise when the image is transformed back into the spatial domain through an inverse Fourier transformation. This step is crucial for enhancing the clarity and readability of the image, paving the way for more accurate annotation and measurement. The next phase, annotation, involves the meticulous marking of the external and internal surfaces of the condensates. Utilizing annotation tools such as CVAT, surfaces are denoted as ellipses, with the internal surface selected manually based on an automatic calibration of the external surface. This step is fundamental in defining the regions of interest for subsequent analysis, ensuring that measurements are conducted with precision. The final step in the analysis process is the measurement of relative signal strength within the condensates. By generating 20 intermediate ellipses through linear interpolation

between the annotated internal and external surfaces, researchers can systematically count the area-normalized signals within each segmented region. This meticulous approach allows for the quantification of signal strength across the condensate, from the internal to the external surface. The data is then plotted with the normalized distance from the internal surface on the x-axis, providing a comprehensive visualization of the signal distribution within the condensate.

2. Protein sequences in Table 3

Table 3. All amino acid sequences of protein used in this work.

Protein	Amino acid Sequence
FUS-ERG	MASNDYTQQATQSYGAYPTQPGQGYSQQSSQPYGQQSY<br>SGYSQSTDTSGYGQSSYSSYGQSQNTGYGTQSTPQGYGS<br>TGGYGSSQSSQSSYGQQSSYPGYGQQPAPSSTSGSYGS<br>SSQSSSYGQPQSGSYSQQPSYGGQQQSYGQQQSYNPPQ<br>GYGQQNQYNSSSGGGGGGGGGNYGQDQSSMSSGGGS<br>GGGYGNQDQSGGGGSGGYGQQDRGGRGRGGSGGGGG<br>GGGGGYNRSSGGYEPRGRGGGRGGRGGMGGSDRGGFN<br>KFGGSGQIQLWQFLLELLSDSSNSSCITWEGTNGEFKMTD<br>PDEVARRWGERKSKPNMNYDKLSRALRYYYDKNIMTKVH<br>GKRYAYKFDFHGIAQALQPHPPESSLYKYPSDLPYMGSYH<br>AHPQKMNFVAPHPPALPVTSSSFFAAPNPYWNSPTGGIYP<br>NTRLPTSHMPSHLGTYY*
FUS-ERG<br>9YS	MRMASNDYTQQATQSYGAYPTQPGQGYSQQSSQPYGQQ<br>SYSGYSQSTDTSGYGQSSYSSYGQSQNTGYGTQSTPQGY<br>GSTGGYGSSQSSQSSYGQQSSYPGYGQQPAPSSTSGSY<br>GSSSQSSSYGQPQSGSSSQQPSSGGQQQSSGQQQSSNP<br>PQGSGQQNQSNSSSGGGGGGGGGNSGQDQSSMSSGG<br>GSGGGSGNQDQSGGGGSGGSGQQDRGGRGRGGSGGG<br>GGGGGGGSNRSSGGSEPRGRGGGRGGRGGMGGSDRG<br>GFNKMFGGSGQIQLWQFLLELLSDSSNSSCITWEGTNGEF<br>KMTDPDEVARRWGERKSKPNMNYDKLSRALRYYYDKNIM<br>TKVHGKRYAYKFDFHGIAQALQPHPPESSLYKYPSDLPYM<br>GSYHAHPQKMNFVAPHPPALPVTSSSFFAAPNPYWNSPTG<br>GIYPNTRLPTSHMPSHLGTYY*
FUS-ERG<br>27YS	MRMASNDSTQQATQSSGASPTQPGQGSSQQSSQPSGQQ<br>SSSGSSQSTDTSGSGQSSSSSSGQSQNTGSGTQSTPQGS<br>GSTGGSGSSQSSQSSSGQQSSSPGSGQQPAPSSTSGSS

	GSSSQSSSSGQPQSGSSSQQPSSGGQQQSSGQQQSSNP<br>PQGSGQQNQSNSSSGGGGGGGGGGNSGQDQSSMSSGG<br>GSGGGSGNQDQSGGGGSGGSGQQDRGGRGRGGSGGG<br>GGGGGGGSNRSSGGSEPRGRGGGRGGRGGMGGSDRG<br>GFNKMFGGSGQIQLWQFLLELLSDSSNSSCITWEGTNGEF<br>KMTDPDEVARRWGERKSKPNMNYDKLSRALRYYYDKNIM<br>TKVHGKRYAYKFDFHGIAQALQPHPPESSLYKYPSDLPYM<br>GSYHAHPQKMNFVAPHPPALPVTSSSFFAAPNPYWNSPTG<br>GIYPNTRLPTSHMPSHLGTYY*
FUS-ERG<br>9RA	MASNDYTQQATQSYGAYPTQPGQGYSQQSSQPYGQQSY<br>SGYSQSTDTSGYGQSSYSSYGQSQNTGYGTQSTPQGYGS<br>TGGYGSSQSSQSSYGQQSSYPGYGQQPAPSSTSGSYGS<br>SSQSSSYGQPQSGSYSQQPSYGGQQQSYGQQQSYNPPQ<br>GYGQQNQYNSSSGGGGGGGGGGNYGQDQSSMSSGGGS<br>GGGYGNQDQSGGGGSGGYGQQDAGGAGAGGSGGGGG<br>GGGGGYNASSGGYEPAGAGGGAGGAGGMGGSDAGGFN<br>KFGGSGQIQLWQFLLELLSDSSNSSCITWEGTNGEFKMTD<br>PDEVARRWGERKSKPNMNYDKLSRALRYYYDKNIMTKVH<br>GKRYAYKFDFHGIAQALQPHPPESSLYKYPSDLPYMGSYH<br>AHPQKMNFVAPHPPALPVTSSSFFAAPNPYWNSPTGGIYP<br>NTRLPTSHMPSHLGTYY*
FUS-ERG<br>5RA	MASNDYTQQATQSYGAYPTQPGQGYSQQSSQPYGQQSY<br>SGYSQSTDTSGYGQSSYSSYGQSQNTGYGTQSTPQGYGS<br>TGGYGSSQSSQSSYGQQSSYPGYGQQPAPSSTSGSYGS<br>SSQSSSYGQPQSGSYSQQPSYGGQQQSYGQQQSYNPPQ<br>GYGQQNQYNSSSGGGGGGGGGGNYGQDQSSMSSGGGS<br>GGGYGNQDQSGGGGSGGYGQQDAGGAGAGGSGGGGG<br>GGGGGYNRSSGGYEPRGRGGGRGGAGGMGGSDAGGFN<br>KFGGSGQIQLWQFLLELLSDSSNSSCITWEGTNGEFKMTD<br>PDEVARRWGERKSKPNMNYDKLSRALRYYYDKNIMTKVH<br>GKRYAYKFDFHGIAQALQPHPPESSLYKYPSDLPYMGSYH<br>AHPQKMNFVAPHPPALPVTSSSFFAAPNPYWNSPTGGIYP<br>NTRLPTSHMPSHLGTYY*
FUS-Gal4	MASNDYTQQATQSYGAYPTQPGQGYSQQSSQPYGQQSY<br>SGYSQSTDTSGYGQSSYSSYGQSQNTGYGTQSTPQGYGS<br>TGGYGSSQSSQSSYGQQSSYPGYGQQPAPSSTSGSYGS<br>SSQSSSYGQPQSGSYSQQPSYGGQQQSYGQQQSYNPPQ<br>GYGQQNQYNSSSGGGGGGGGGGNYGQDQSSMSSGGGS<br>GGGYGNQDQSGGGGSGGYGQQDRGGRGRGGSGGGGG<br>GGGGGYNRSSGGYEPRGRGGGRGGRGGMGGSDRGGFN<br>KFGKLLSSIEQACDICRLKKLKCSKEKPKCAKCLKNNWECR<br>YSPKTKRSPLTRAHLTEVESRLERLEQLFLLIFPREDLDMILK<br>MDSLQDIKALLTGLFVQDNVNKDAVTDRLASVETDMPLTLR<br>QHRISATSSSEESSNKGQRQLTVS*
FUS-Gal4<br>no RGG	MASNDYTQQATQSYGAYPTQPGQGYSQQSSQPYGQQSY<br>SGYSQSTDTSGYGQSSYSSYGQSQNTGYGTQSTPQGYGS<br>TGGYGSSQSSQSSYGQQSSYPGYGQQPAPSSTSGSYGS<br>SSQSSSYGQPQSGSYSQQPSYGGQQQSYGQQQSYNPPQ<br>GYGQQNQYNSSSGGGGGGGGGGNYGQDQSSMSSGGGS<br>GGGYGNQDQSGGGGSGGYGQQDGGSGGSGGSGGSMKL

	LSSIEQACDICRLKKLKCSKEKPKCAKCLKNNWECRYSPKT KRSPLTRAHLTEVESRLERLEQLFLLIFPREDLDMILKMDSL QDIKALLTGLFVQDNVNKDAVTDRLASVETDMPLTLRQHRI SATSSSEESSNKGQRQLTVS*
	MASNDYTQQATQSYGAYPTQPGQGYSQQSSQPYGQQSY SGYSQSTDTSGYGQSSYSSYGQSQNTGYGTQSTPQGYGS TGGYGSSQSSQSSYGQQSSYPGYGQQPAPSSTSGSYGS SSQSSSYGQPQSGSYSQQPSYGGQQQSYGQQQSYNPPQ GYGQQNQYNSSSGGGGGGGGGNYGQDQSSMSSGGGS GGGYGNQDQSGGGGSGGYGQQDRGGRGRGGSGGGGG GGGGGYNRSSGGYEPRGRGGGRGGRGGMGGSDRGGFN KFGGVFKKEVYLHTSPHLKADVLFQTDPTAEMAAESLPFSF GTLSSWELEAWYEDLQEVLSSDENGGTYVSPPGNEEEES KIFTTLDPASLAWLTEEEPEPAEVTSTSQSPHSPDSSQSSL AQEEEEEDQGRTRKRKQSGHSPARAGKQRMKEKEQENE RKVAQLAEENERLKQEIERLTREVEATRRALIDRMVNLHQA
FUS-DDIT3 9RA	MASNDYTQQATQSYGAYPTQPGQGYSQQSSQPYGQQSY SGYSQSTDTSGYGQSSYSSYGQSQNTGYGTQSTPQGYGS TGGYGSSQSSQSSYGQQSSYPGYGQQPAPSSTSGSYGS SSQSSSYGQPQSGSYSQQPSYGGQQQSYGQQQSYNPPQ GYGQQNQYNSSSGGGGGGGGGNYGQDQSSMSSGGGS GGGYGNQDQSGGGGSGGYGQQDAGGAGAGGSGGGGG GGGGGYNASSGGYEPAGAGGGAGGAGGMGGSDAGGFN KFGGVFKKEVYLHTSPHLKADVLFQTDPTAEMAAESLPFSF GTLSSWELEAWYEDLQEVLSSDENGGTYVSPPGNEEEES KIFTTLDPASLAWLTEEEPEPAEVTSTSQSPHSPDSSQSSL AQEEEEEDQGRTRKRKQSGHSPARAGKQRMKEKEQENE RKVAQLAEENERLKQEIERLTREVEATRRALIDRMVNLHQA

3. Oligo Preparation

All oligo sequences were reported below, and the relevant DNA binding sites were highlighted in red:

1× Gal4DBD binding site Top (25-bp): 5' – ATA T – *CGG AGG ACA GTC CTC CG* – AAT A – 3'

1× Gal4DBD binding site Bottom (25-bp): 5' – TAT TCG GAG GAC TGT CCT CCG ATA T – 3'

2× GGAA binding site Top (25-bp): 5' – CCG GAC AT *GGA AGG AA*G GCA CTC CA – 3'

2× GGAA binding site Bottom (25-bp): 5' – TGG AGT GCC TTC CTT CCA TGT CCG G – 3'

4× GGAA binding site Top (25-bp): 5' – CCG G *GGA AGG AAG GAA GGA A* CTC CA – 3'

4× GGAA binding site Bottom (25-bp): 5' –TGG AGT TCC TTC CTT CCT TCC CCG G – 3'

Control sequence Top (25-bp): 5' –ATA TAT TTT TTA CAA TAG AAT AAT A – 3'

Control sequence Bottom (25-bp): 5' –TAT TAT TCT ATT GTA AAA AAT ATA T – 3'

Microsatellite DNA (306-bp, 25× GGAA repeats): 5' – GTC GAC TAG GTT TTC CTC TTA TGC TGA GAA TTC CAG GTC CTG GAG AAG AAG AAA AAG AGA AAG AAA GAG AGA GAG AGA AGG AGT GAG AGA GGG AGG GAG GGA GGG AGG GAG GGA – *GGAA GGAA GGAA GGAA GGAA GGAA GGAA AGGAA GGAA GGAA GGAA GGAA GGAA GGAA AGGAA GGAA GGAA GGAA GGAA GGAA GGAA GGAA GGAA GGAA GGAA* – AAG AAA CAG CAA AAA AAG AAA GAG GGA GGA TGG GAG GGA GGG AAA AAG TAA AAA TGA TTC TGT ATC AGC TGG TAT ATA CCA ACA ACT AGT – 3'

4. Supplementary Figures

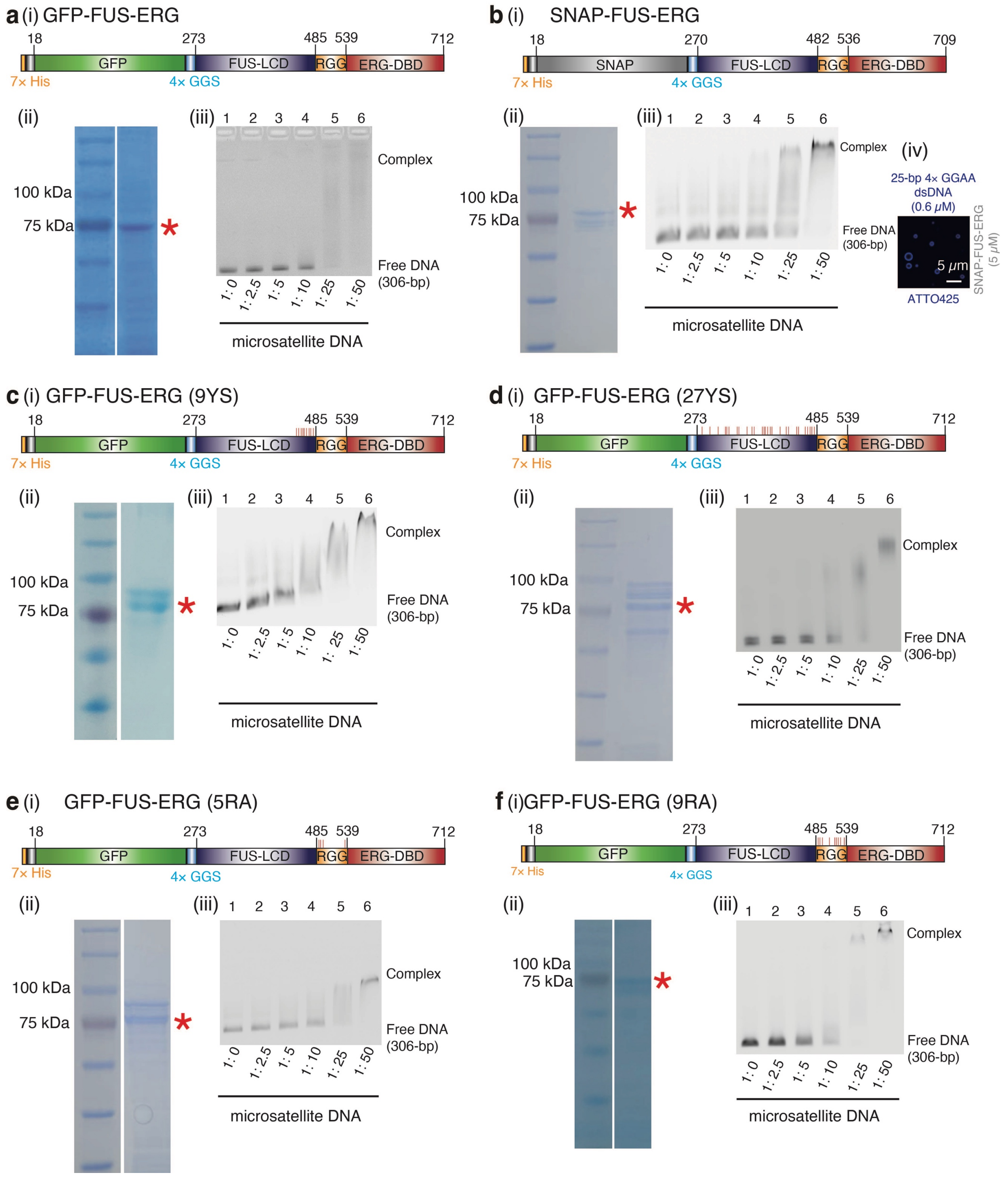

Supplementary Fig. 1. In vitro purified FET fusion proteins in this work. (a-f) (i) Schematic; (ii) SDS-PAGE; (iii) EMSA (1.2% agarose gel). GFP-FUS-ERG (a), SNAP-FUS-ERG (b), GFP-FUS-ERG (9YS) (c), GFP-FUS-ERG (27YS) (d), GFP-FUS-ERG (5RA) (e), GFP-FUS-ERG (9RA) (f). b(iv) 5 μM SNAP-FUS-ERG mixed with 10 ng/μL 0.6 μM 25-bp 4× GGAA dsDNA labeled with ATTO425. Scale bar: 5μm in b(iv).

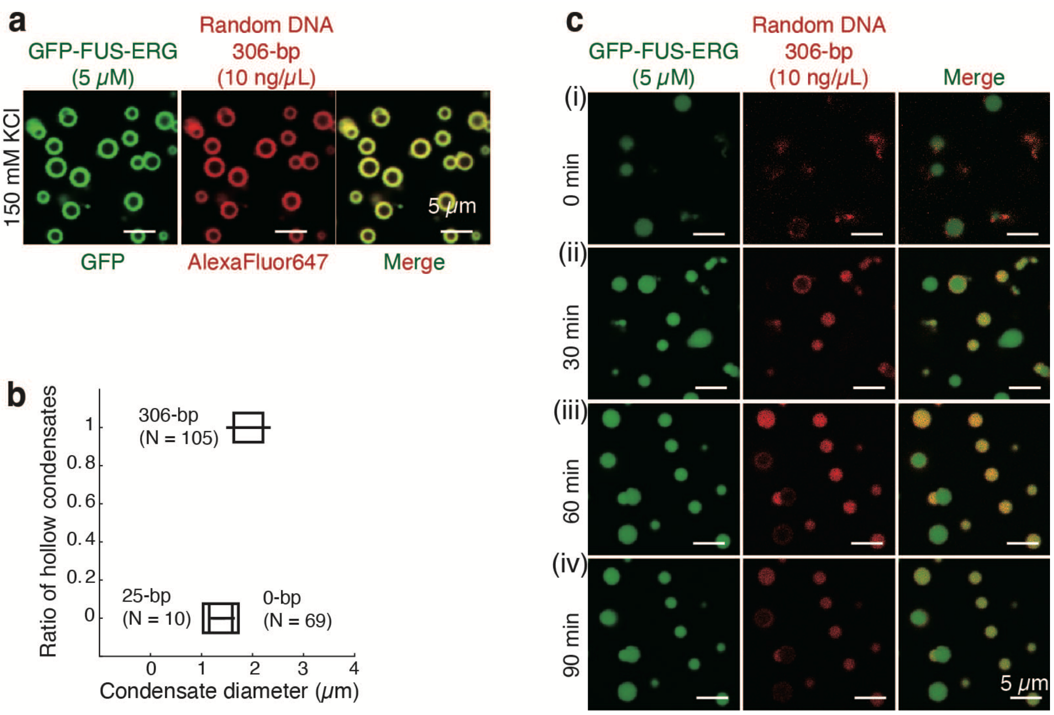

Supplementary Fig. 2. In vitro droplet experiments indicate that GFP-FUS-ERG could form hollow co-condensate with 306-bp random dsDNA. (**a**) 5 μM GFP-FUS-ERG mixed with 10 ng/μL 306-bp AlexaFluor647-labeled random dsDNA. (**b**) The percentage of hollow co-condensates versus condensate diameter for the condition: 5 μM GFP-FUS-ERG (Fig. 1a), 5 μM GFP-FUS-ERG and 10 ng/μL (0.6 μM) 25-bp AlexaFluor647-labeled random dsDNA (Fig. 1b(i)), and 5 μM GFP-FUS-ERG and 10 ng/μL 306-bp AlexaFluor647-labeled random dsDNA (a). Error bars, mean ± s.d. All in vitro droplet assays were executed under physiological conditions, specifically 40 mM Tris-HCl (pH = 7.5), 150 mM KCl, 2 mM $MgCl_2$, 1 mM DTT and 0.2 mg/mL BSA, with thorough mixing and a 30-minute incubation period prior to imaging, unless otherwise indicated. (**c**) Time course of hollow co-condensate formation of 5 μM GFP-FUS-ERG mixed with 10 ng/μL 306-bp AlexaFluor647-labeled random dsDNA at 0-min (i), 30-min (ii), 60-min (iii), and 90-min (iv). At the 0-minute time point, we injected dsDNA. Scale bar: 5 μm in a and c. Source data are provided as a Source Data file.

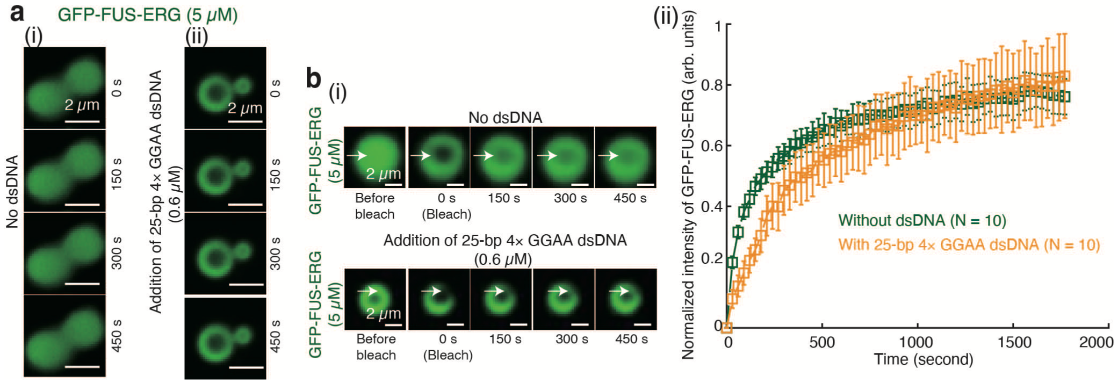

Supplementary Fig. 3. Both hollow and homogeneous condensates exhibited slow fusion kinetics. (**a**) Time course of the fusion event between homogeneous condensates of GFP-FUS-ERG (i), or hollow co-condensates of GFP-FUS-ERG and 25-bp 4× GGAA dsDNA (ii). (**b**) (i) FRAP experiment of d; (ii) FRAP curves. Green, e(i); Orange, e(ii). Independent FRAP experiments were repeated: e(i), N = 10; e(ii), N = 10. Error bars, mean ± s.d. All in vitro droplet assays were executed under physiological conditions, specifically 40 mM Tris-HCl (pH = 7.5), 150 mM KCl, 2 mM $MgCl_2$, 1 mM DTT and 0.2 mg/mL BSA, with thorough mixing and a 30-minute incubation period prior to imaging, unless otherwise indicated. Scale bar: 2 μm in a and b. Source data are provided as a Source Data file.

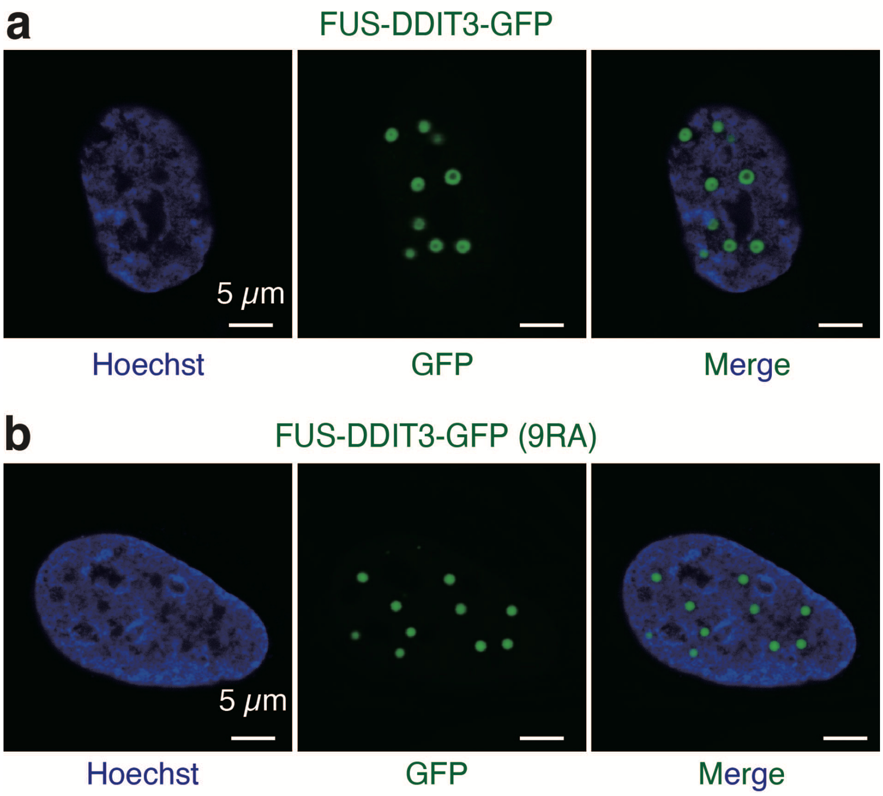

Supplementary Fig. 4. The hollow co-condensates of FET fusion oncoproteins in vivo. (**a**) FUS-DDIT3-GFP hollow co-condensates in the nucleus; (**b**) FUS-DDIT3-GFP (9RA) formed homogeneous condensates in the nucleus. FUS-DDIT3-GFP or FUS-DDIT3-GFP (9RA) was overexpressed in U2OS cells. Hoechst was used to stain the DNA molecules in the nucleus (blue color). Scale bar: 5 μm in and b.

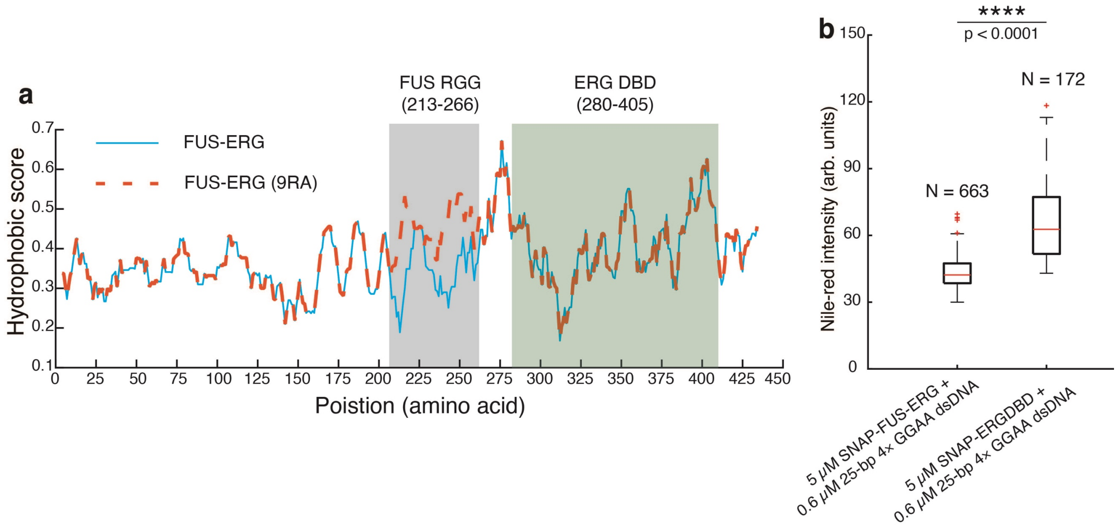

Supplementary Fig. 5. Hydropathicity analysis of FUS-ERG. (**a**) Hydrophobic score prediction of FUS-ERG and FUS-ERG (9RA), the window size of amino acids is 9, and the scale is normalized from 0 to 1. The higher score represents the higher hydrophobicity [14]. (**b**) Boxplot of the nile-red intensity of 5 μM SNAP-FUS-ERG mixed with 0.6 μM 25-bp 4× GGAA dsDNA, and 5 μM SNAP-ERGDBD mixed with 0.6 μM 25-bp 4× GGAA dsDNA. The total number N examined over one-time in vitro droplet experiments. The condensates were stained by nile-red under a room temperature, and the excitation wavelength is 553 nm. All experiments were conducted in the buffer containing 40 mM Tris-HCl (pH = 7.5), 150 mM KCl, 2 mM $MgCl_2$, 1 mM DTT and 0.2 mg/mL BSA. For the boxplot, the red bar represents median. The bottom edge of the box represents 25th percentiles, and the top is 75th percentiles. Most extreme data points are covered by the whiskers except outliers. The '+' symbol is used to represent the outliers. Statistical significance was analyzed using unpaired t test for two groups. P value: two-tailed; p value style: GP: 0.1234 (ns), 0.0332 (*), 0.0021 (**), 0.0002 (***), <0.0001 (****). Exact P values are as follows: SNAP-FUS-ERG vs. SNAP-ERGDBD, P < 0.0001. Confidence level: 95%. Source data are provided as a Source Data file.

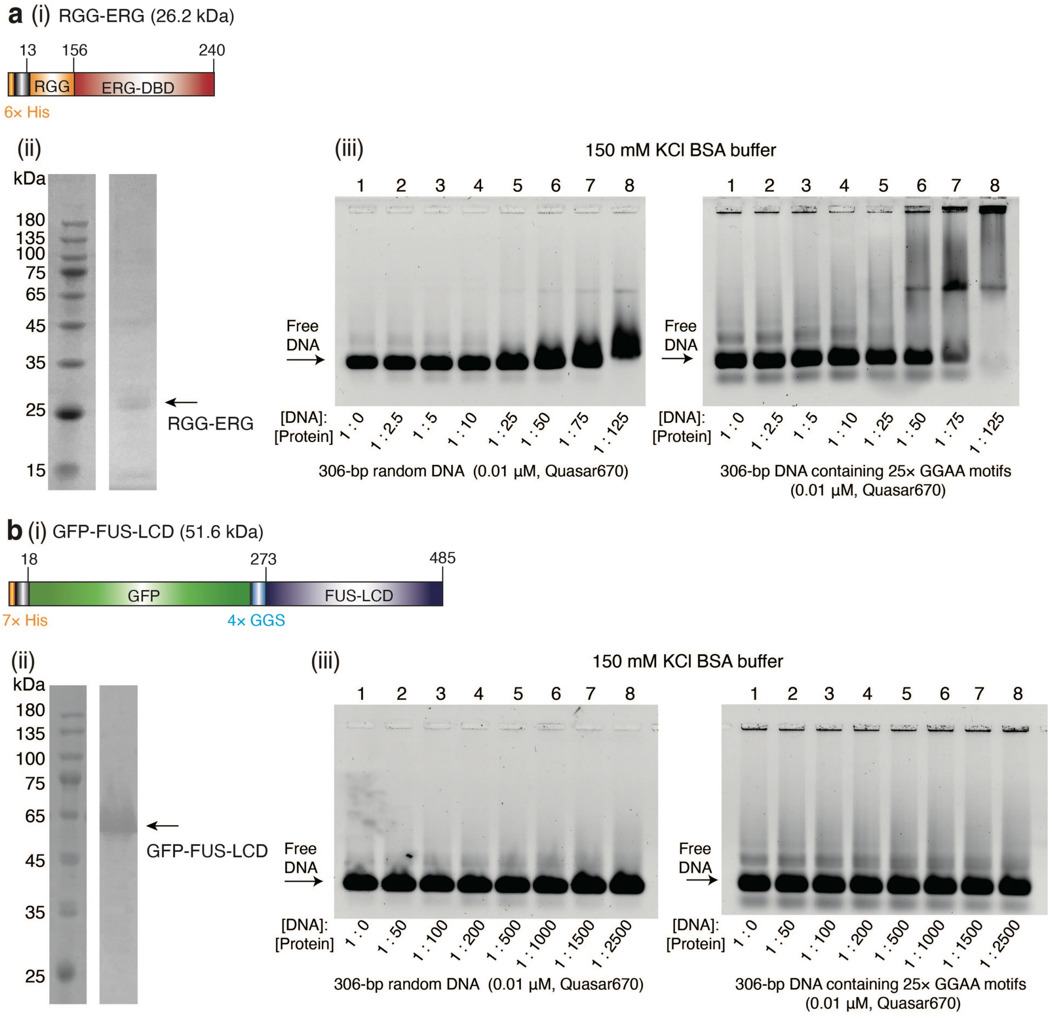

Supplementary Fig. 6. Biochemical assays for RGG-ERG and GFP-FUS-LCD. In vitro purified RGG-ERG (a) and GFP-FUS-LCD (b). (i) Schematic representation of the protein constructs; (ii) SDS-PAGE analysis confirming protein purity; (iii) Electrophoretic mobility shift assay (EMSA) performed on a 1.2% agarose gel. DNA substrates were labeled with Quasar670 and imaged using an Amersham Typhoon RGB system (635 nm excitation, Cy5 670BP30 emission filter).

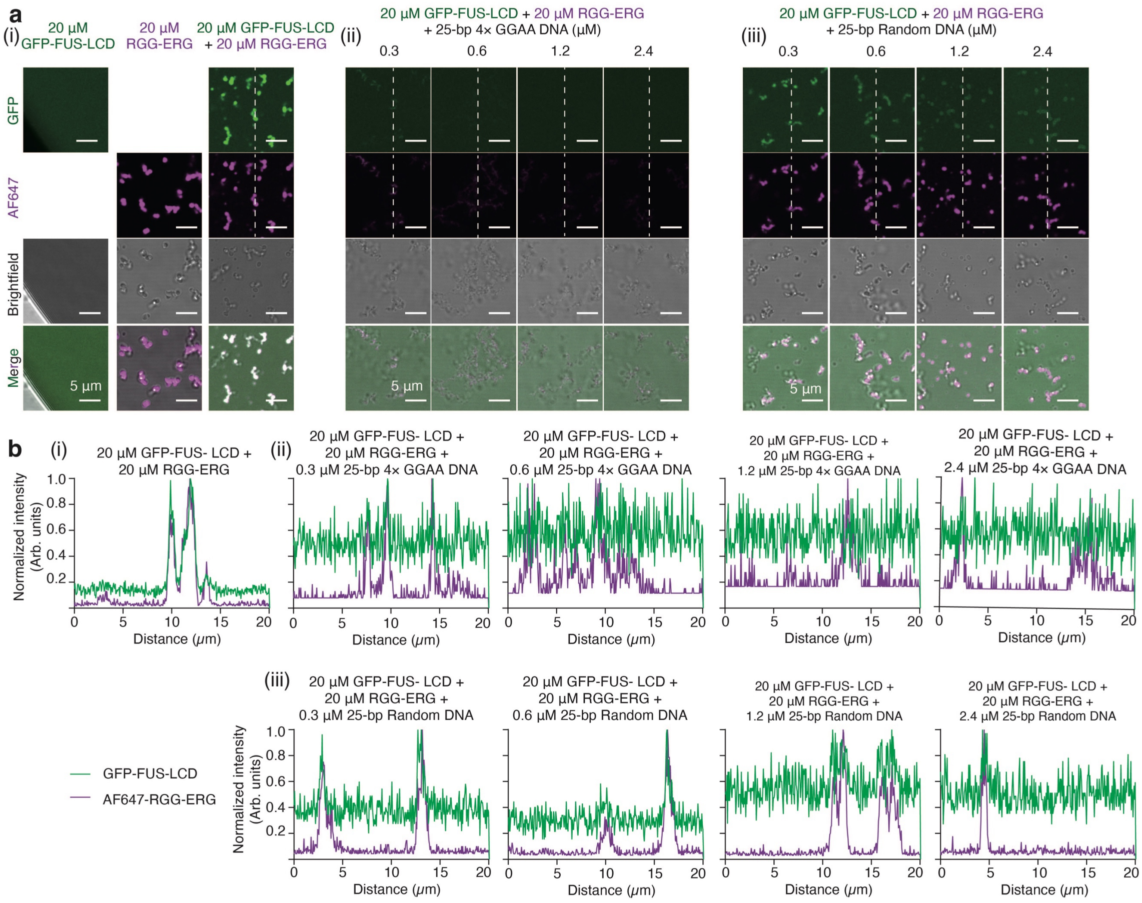

Supplementary Fig. 7. High concentration of dsDNA containing GGAA motifs can inhibit the interaction between GFP-FUS-LCD and RGG-ERG. (a) In vitro droplet assays of GFP-FUS-LCD, RGG-ERG, and mixtures of the two proteins with or without dsDNA. (i) 20 µM GFP-FUS-LCD only, 20 µM RGG-ERG only and 20 µM GFP-FUS-LCD mixed with 20 µM RGG-ERG. (ii)-(iii) The addition of varying concentrations of 25-bp 4□ GGAA DNA (ii) or 25-bp Random DNA (iii) to a system containing 20 µM GFP-FUS-LCD mixed with 20 µM RGG-ERG. (b) Normalized intensity profiles from conditions in (a). (i) 20 µM GFP-FUS-LCD mixed with 20 µM RGG-ERG. (ii)-(iii) Conditions containing 20 µM GFP-FUS-LCD, 20 µM RGG-ERG and varying concentrations of 25-bp 4× GGAA DNA (ii) or 25-bp Random DNA (iii). In this set of experiments, RGG-ERG was prepared by mixing unlabeled protein with AlexaFluor647-labeled protein at a ratio of 100:1. In all experimental conditions, the

proteins and DNA were first mixed, and then imaged after incubation at room temperature for 30 minutes. The working buffer containing 40 mM Tris-HCl (pH 7.5), 150 mM KCl, 2 mM MgCl2, 1 mM DTT and 0.2 mg/mL BSA.

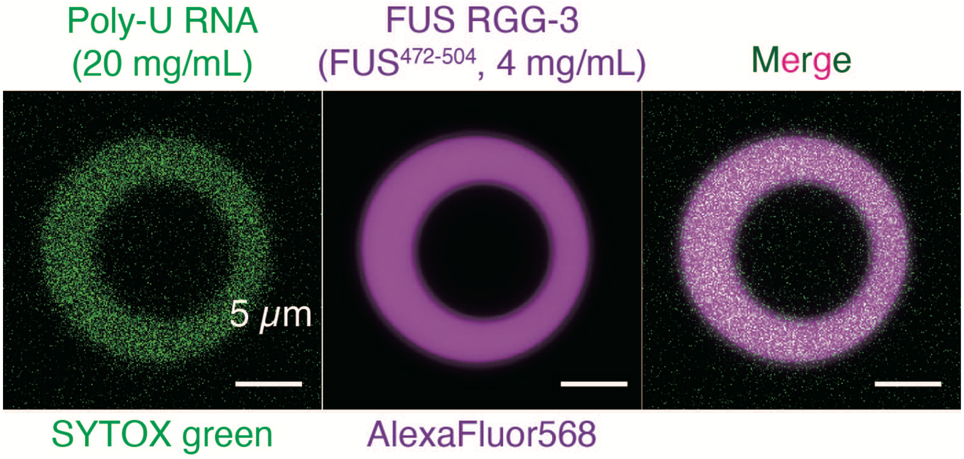

Supplementary Fig. 8. In vitro droplet experiments indicate that FUS RGG motif can form hollow co-condensates with RNA. 4 mg/mL AlexaFluor568 labeled FUS RGG-3 (FUS No. 471-504) mixed with 20 mg/mL SYTOX Green (ThermoFisher, Cat. S7020) stained Poly U RNA (Sigma-Aldrich, Cat. 27416-86-0). Scale bar: 5 μm.

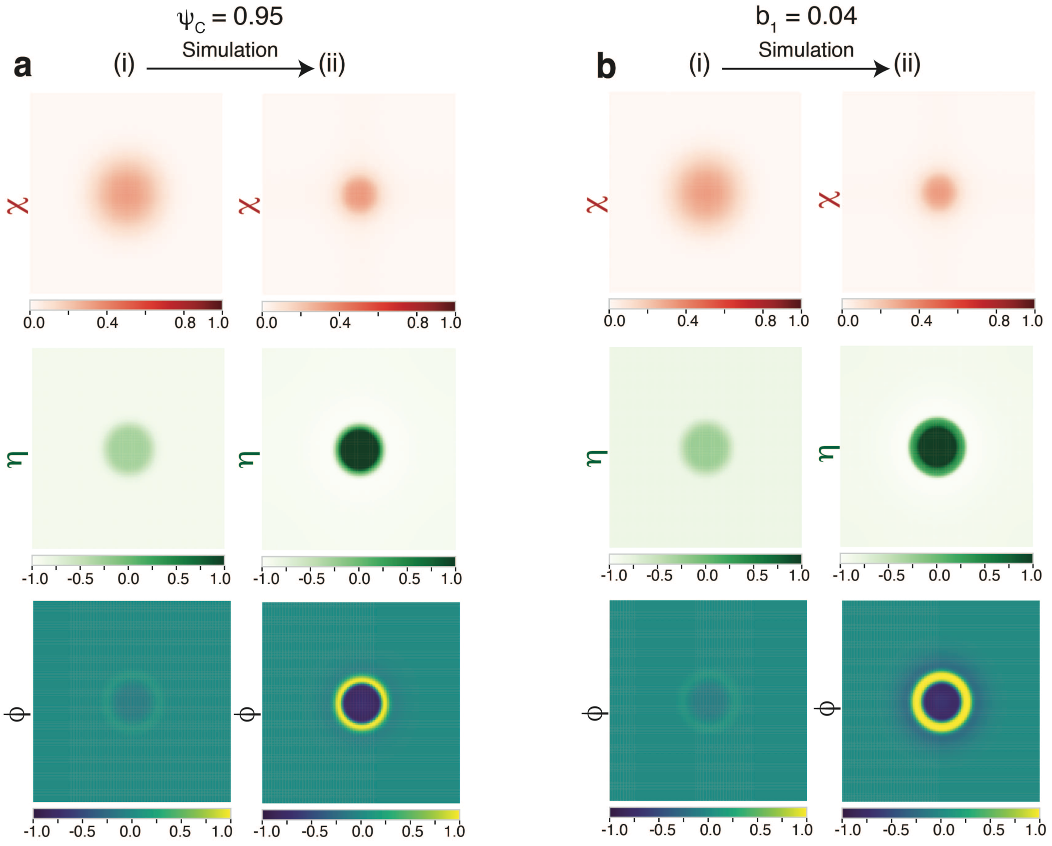

Supplementary Fig. 9. Mathematical model under different model coefficients. Order parameters denoting protein-DNA complex concentration (η), dsDNA concentration (χ) and hydrophobic and hydrophilic distributions within condensates (ϕ) are represented. (**a**) Simulation under higher $\psi_C = 0.95$. (i) Initial value of simulation (ii) Under higher ψ_C, only homogeneous condensate is observed. (**b**) Simulation under lower $b_1 = 0.04$. (i) Initial value of simulation (ii) Under lower b_1 only homogeneous condensate is observed.

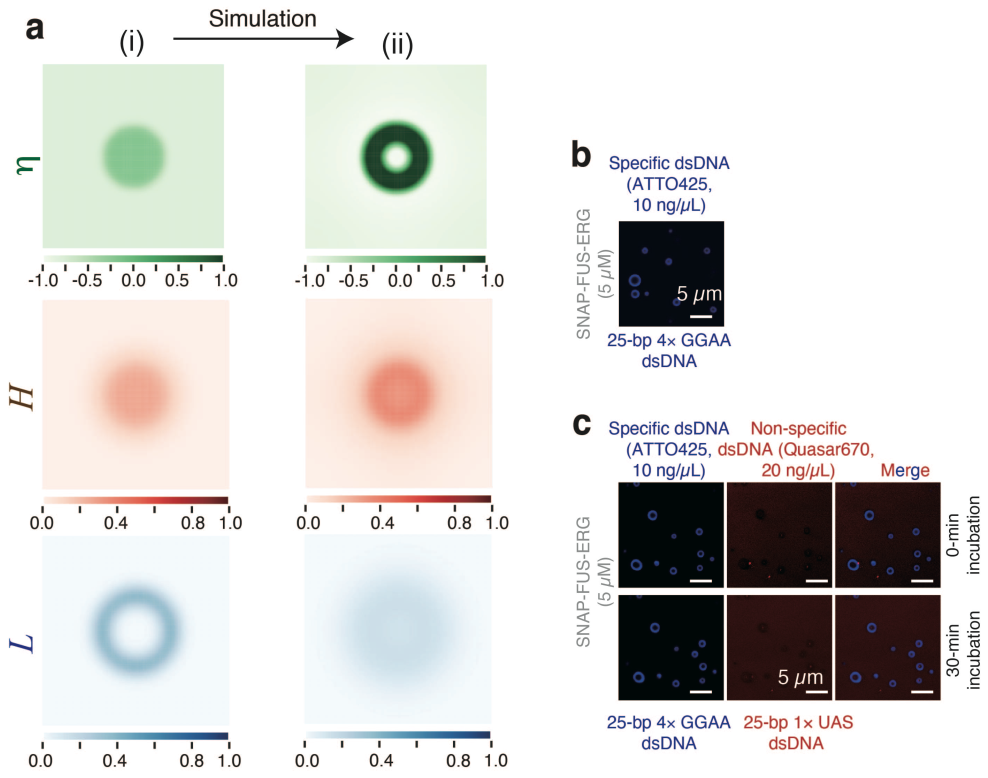

Supplementary Fig. 10. Numerical simulation and in vitro droplet experiments indicate that the hollow co-condensates can block non-specific dsDNA. (**a**) Order parameters denote protein-DNA complex concentration (η) and DNA concentration (High affinity to protein H (red); Low affinity to protein L (blue)) are represented. (**a**) Simulation where high affinity DNA is first introduced H (i) Initial values of simulation, H is first diffused with proteins while L is set around the droplet. (ii) Equilibrium of simulation, hollow structure come up with H distributed across the structure, DNA with low affinity L fails to enter the condensates. (**b**) 5 μM SNAP-FUS-ERG mixed with 10 ng/μL (0.6 μM) of specific dsDNA (25-bp 4× GGAA dsDNA labeled with ATTO425). (**c**) we injected 20 ng/μL of non-specific dsDNA (25-bp 1× UAS dsDNA labeled with Quasar670) into the sample in a. 0-min incubation and 30-min incubation. Scale bar: 5 μm in b and c.

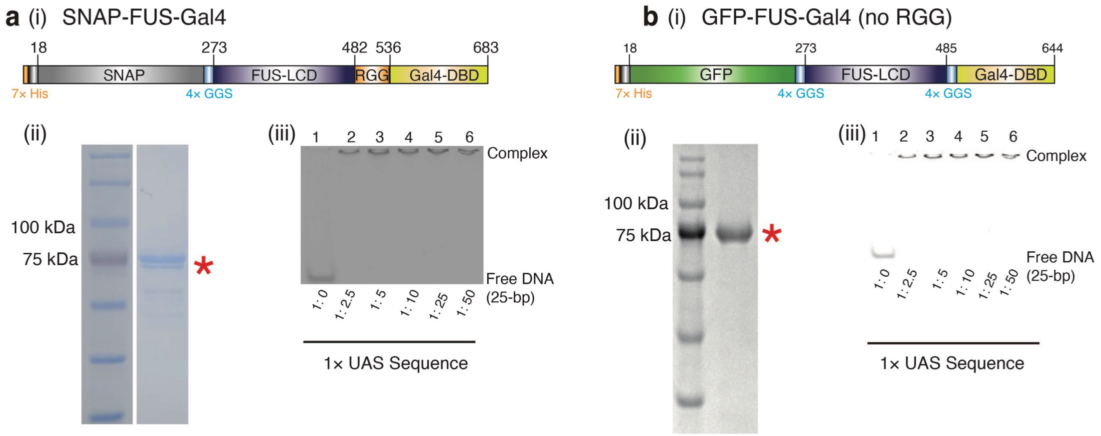

Supplementary Fig. 11. In vitro purified FET fusion proteins in this work. (a-b) (i) Schematic; (ii) SDS-PAGE; (iii) EMSA (1.2% agarose gel). SNAP-FUS-Gal4 (a) and SNAP-FUS-Gal4 (no RGG) (b).

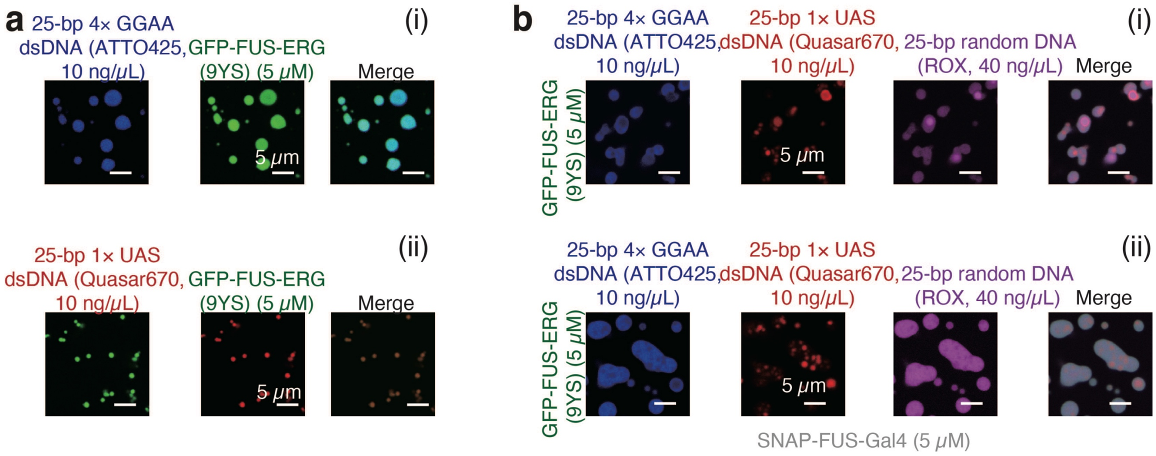

Supplementary Fig. 12. Hollow condensate architecture is necessary for selective dsDNA sorting. (**a**) Fluorescence images of 5 μM GFP-FUS-ERG (9YS) mixed with 10 ng/μL (0.6 μM) ATTO425-labeled 25-bp 4× GGAA dsDNA (i), or 10 ng/μL Quasar670-labeled 25-bp 1× UAS dsDNA (ii). (**b**) Phase separation-based DNA selection. First, 5 μM SNAP-FUS-ERG (9YS) was added into a dsDNA library containing 10 ng/μL (0.6 μM) ATTO425-labeled 25-bp 4× GGAA dsDNA, 10 ng/μL Quasar670-labeled 25-bp 1× UAS dsDNA, and 40 ng/μL ROX-labeled 25-bp random dsDNA. Fluorescence images were shown in (i). Second, 5 μM SNAP-FUS-Gal4 was added into the system, and the fluorescence images were shown in (ii). All experiments were conducted in the buffer containing 40 mM Tris-HCl (pH = 7.5), 150 mM KCl, 2 mM $MgCl_2$, 1 mM DTT and 0.2 mg/mL BSA. Scale bar: 5 μm.

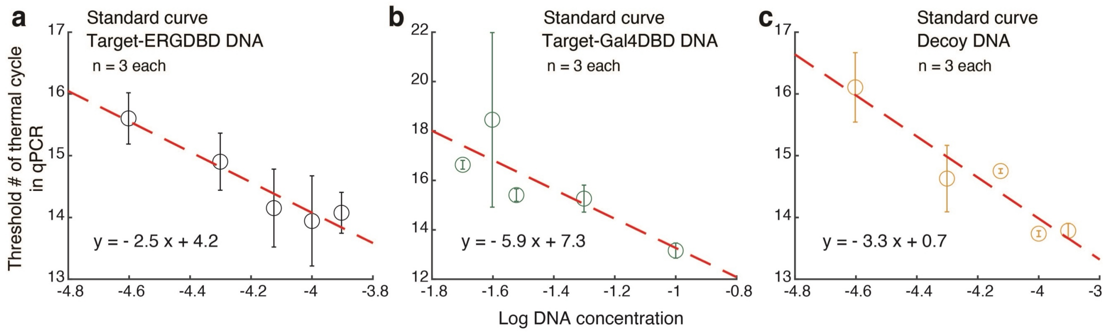

Supplementary Fig. 13. qPCR calibration for Phase separation-based DNA selection. Calibration curves display 5 serial dilution points of DNA library containing target-ERGDBD dsDNA, target-Gal4DBD dsDNA, and decoy dsDNA, and cover dsDNA quantities from 0.000025 to 0.000125 ng. (**a**) the experimental results for target-ERGDBD dsDNA. (**b**) the experimental results for target-Gal4DBD dsDNA. (**c**) the experimental results for decoy dsDNA. The straight red dash lines were used to fit (a)-(c). Source data are provided as a Source Data file.

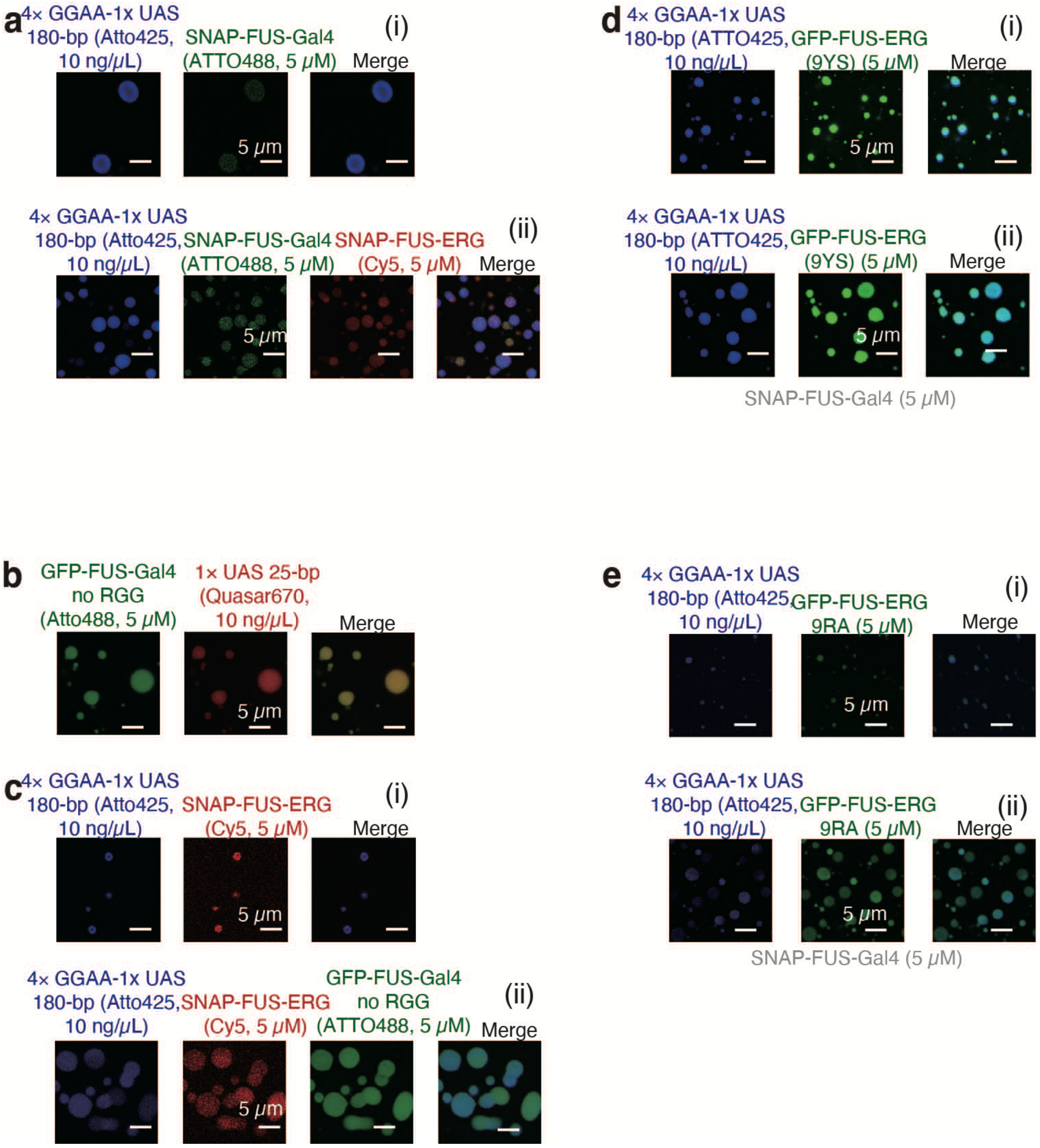

Supplementary Fig. 14. Control experiments for phase separation-based dynamic and hierarchical data selection. (**a**) The experiment in Fig. 5d was repeated, but SNAP-FUS-Gal4 was added firstly (i), and SNAP-FUS-ERG was added secondly (ii). (**b**) Fluorescence images of 5 μM GFP-FUS-Gal4 (no RGG) mixed with 10 ng/μL Quasar670-labeled 25-bp 1× UAS dsDNA. (**c-e**) The experiment in Fig. 5d was repeated, but SNAP-FUS-ERG was added firstly c(i), and GFP-FUS-Gal4 (no RGG) was added secondly c(ii); GFP-FUS-ERG (9YS) was added firstly d(i), and

SNAP-FUS-Gal4 was added secondly d(ii); GFP-FUS-ERG (9RA) was added firstly e(i), and SNAP-FUS-Gal4 was added secondly e(ii). Scale bar: 5 μm.

5. Uncropped scans of all gels in Supplementary Figures

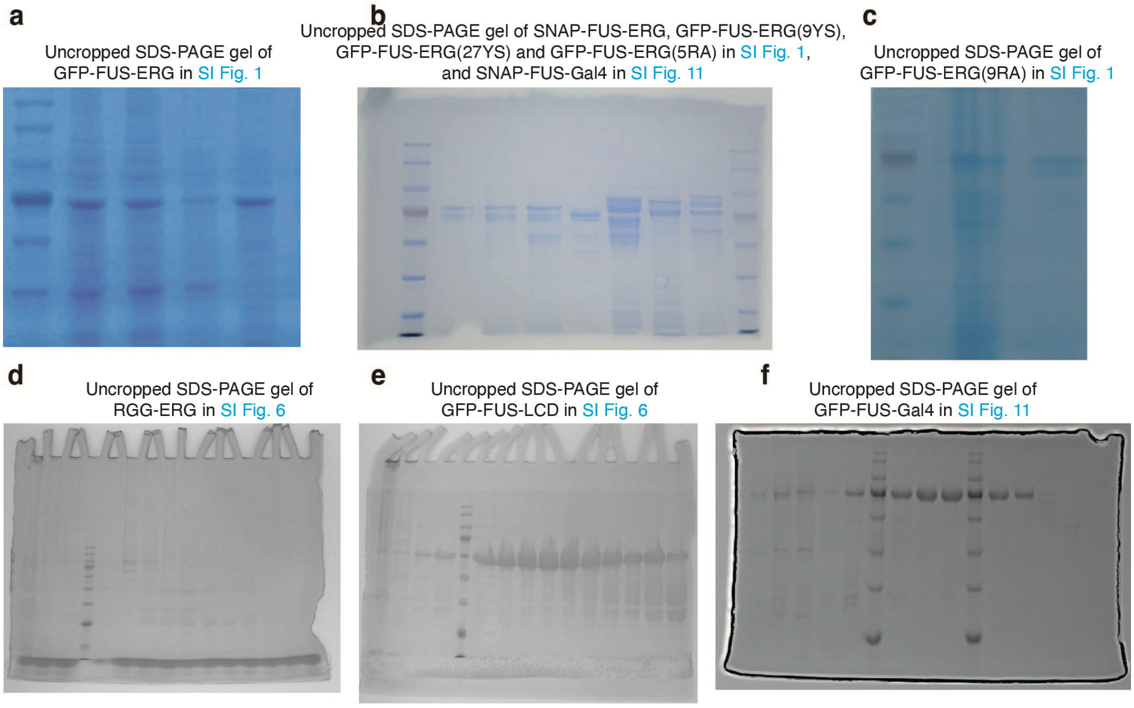

Supplementary References

1 Li, C. *et al.* Deciphering the molecular mechanism underlying morphology transition in two-component DNA-protein cophase separation. *Structure* **33**, 62-77.e68, doi:10.1016/j.str.2024.10.026 (2025).

2 Alshareedah, I., Moosa, M. M., Raju, M., Potoyan, D. A. & Banerjee, P. R. Phase transition of RNA-protein complexes into ordered hollow condensates. *P Natl Acad Sci USA* **117**, 15650-15658, doi:10.1073/pnas.1922365117 (2020).

3 Gasior, K. *et al.* Partial demixing of RNA-protein complexes leads to intradroplet patterning in phase-separated biological condensates. *Phys Rev E* **99**, doi:ARTN 012411 10.1103/PhysRevE.99.012411 (2019).

4 Lao, Z. H. *et al.* Insights into the Atomistic Mechanisms of Phosphorylation in Disrupting Liquid-Liquid Phase Separation and Aggregation of the FUS Low Domain. *J Chem Inf Model*, doi:10.1021/acs.jcim.2c00414 (2022).

5 Paloni, M., Bailly, R., Ciandrini, L. & Barducci, A. Unraveling Molecular Interactions in Liquid-Liquid Phase Separation of Disordered Proteins by Atomistic Simulations. *J Phys Chem B* **124**, 9009-9016, doi:10.1021/acs.jpcb.0c06288 (2020).

6 Rauscher, S. & Pomès, R. The liquid structure of elastin. *Elife* **6**, doi:ARTN e26526 10.7554/eLife.26526 (2017).

7 Mao, S., Chakraverti-Wuerthwein, M. S., Gaudio, H. & Kosmrlj, A. Designing the Morphology of Separated Phases in Multicomponent Liquid Mixtures. *Phys Rev Lett* **125**, doi:ARTN 218003 10.1103/PhysRevLett.125.218003 (2020).
8 Zhang, R. Y., Mao, S. & Haataja, M. P. Chemically reactive and aging macromolecular mixtures I: Phase diagrams, spinodals, and gelation. *J Chem Phys* **160**, doi:Artn 244903 10.1063/5.0196793 (2024).
9 Ito, A. Domain patterns in copolymer-homopolymer mixtures. *Phys Rev E* **58**, 6158-6165, doi:DOI 10.1103/PhysRevE.58.6158 (1998).
10 Ohta, T. & Ito, A. Dynamics of Phase-Separation in Copolymer-Homopolymer Mixtures. *Phys Rev E* **52**, 5250-5260, doi:DOI 10.1103/PhysRevE.52.5250 (1995).
11 Han, Y. C., Xu, Z. R., Shi, A. C. & Zhang, L. Pathways connecting two opposed bilayers with a fusion pore: a molecularly-informed phase field approach. *Soft Matter* **16**, 366-374, doi:10.1039/c9sm01983a (2020).
12 Tao, K. *et al.* Tuning Cell Motility via Cell Tension with a Mechanochemical Cell Migration Model. *Biophys J* **118**, 2894-2904, doi:10.1016/j.bpj.2020.04.030 (2020).
13 Tang, T. & Qiao, Z. H. Efficient numerical methods for phase-field equations. *Scientia Sinica Mathematica* **50**, 775-794, doi:10.1360/ssm-2020-0042 (2020).
14 Kyte, J. & Doolittle, R. F. A simple method for displaying the hydropathic character of a protein. *J Mol Biol* **157**, 105-132, doi:https://doi.org/10.1016/0022-2836(82)90515-0 (1982).
