# Supplementary material for: Deciphering the molecular mechanisms of FET fusion oncoprotein–DNA hollow co-condensates
Source: Nat Commun. 2025 Nov 7;16:9823. doi: 10.1038/s41467-025-65069-4 (PMC12594852; doi:10.1038/s41467-025-65069-4)
Supplement: Supplementary file 2 — Description of Additional Supplementary Files [file 41467_2025_65069_MOESM2_ESM.pdf]

Description of Additional Supplementary Files:

Supplementary Movie 1: GFP-FUS-ERG and dsDNA with GGAA microsatellites form spherical shell-like structure in three-dimensions. 5 μM GFP-FUS-ERG and 10 ng/μL (0.6 μM) Quasar670-labeled dsDNA containing 4× GGAA formed spherical hollow co-condensates in vitro, and both proteins and DNA co-localized on the surface of the hollow condensates.

Supplementary Movie 2: dsDNA transfers into GFPFUS-ERG droplet and induces the hollow cocondensate formation. 10 ng/μL (0.6 μM) AlexaFluor647- labeled 25-bp dsDNA containing 4× GGAA was gently added into the pre-formed GFP-FUS-ERG droplet. As shown in the movie, dsDNA firstly transferred into the droplet, and then the proteins started to move to the surface of the condensate together with dsDNA along with the inner surface getting larger and clearer.

Supplementary Movie 3: Simulations for FUS-ERGDNA hollow co-condensate formation. Formation of simulated hollow co-condensates shown in Fig. 4a. Order parameters denoting protein-DNA complex concentration (η), hydrophobic and hydrophilic distributions within condensates (ϕ) and dsDNA concentration (χ) are represented.

Supplementary Movie 4: Simulations for FUS-ERGDNA hollow co-condensate formation under different model coefficient $\boldsymbol{\psi C}$. Formation of simulated hollow cocondensates shown in Supplementary Fig. 9a. Order parameters denoting protein-DNA complex concentration (η), hydrophobic and hydrophilic distributions within condensates (ϕ) and dsDNA concentration (χ) are represented.

Supplementary Movie 5: Simulations for FUS-ERGDNA hollow co-condensate formation under different model coefficient $\boldsymbol{b1}$. Formation of simulated hollow cocondensates shown in Supplementary Fig. 9b. Order parameters denoting protein-DNA complex concentration (η), hydrophobic and hydrophilic distributions within complex (ϕ) and dsDNA concentration (χ) are represented.

Supplementary Movie 6: Simulations for PRM-RNA hollow co-condensate formation. Formation of simulated hollow co-condensates shown in Fig. 4d. Order parameter denoting RNA-protein complex concentration (η), hydrophobic and hydrophilic distributions within complex (ϕ) and RNA concentration (χ) are represented.
