# Supplementary material for: Deciphering the molecular mechanisms of FET fusion oncoprotein–DNA hollow co-condensates
Source: Nat Commun. 2025 Nov 7;16:9823. doi: 10.1038/s41467-025-65069-4 (PMC12594852; doi:10.1038/s41467-025-65069-4)
Supplement: Supplementary file 9 — Reporting Summary [file 41467_2025_65069_MOESM9_ESM.pdf]

nature portfolio

Corresponding author(s): Qi, Zhi

Last updated by author(s): Sep 16, 2025

Reporting Summary

Nature Portfolio wishes to improve the reproducibility of the work that we publish. This form provides structure for consistency and transparency in reporting. For further information on Nature Portfolio policies, see our Editorial Policies and the Editorial Policy Checklist.

Statistics

For all statistical analyses, confirm that the following items are present in the figure legend, table legend, main text, or Methods section.

n/a	Confirmed	
☐	☒	The exact sample size (*n*) for each experimental group/condition, given as a discrete number and unit of measurement
☐	☒	A statement on whether measurements were taken from distinct samples or whether the same sample was measured repeatedly
☐	☒	The statistical test(s) used AND whether they are one- or two-sided<br>*Only common tests should be described solely by name; describe more complex techniques in the Methods section.*
☒	☐	A description of all covariates tested
☐	☒	A description of any assumptions or corrections, such as tests of normality and adjustment for multiple comparisons
☐	☒	A full description of the statistical parameters including central tendency (e.g. means) or other basic estimates (e.g. regression coefficient) AND variation (e.g. standard deviation) or associated estimates of uncertainty (e.g. confidence intervals)
☐	☒	For null hypothesis testing, the test statistic (e.g. *F*, *t*, *r*) with confidence intervals, effect sizes, degrees of freedom and *P* value noted<br>*Give P values as exact values whenever suitable.*
☒	☐	For Bayesian analysis, information on the choice of priors and Markov chain Monte Carlo settings
☒	☐	For hierarchical and complex designs, identification of the appropriate level for tests and full reporting of outcomes
☒	☐	Estimates of effect sizes (e.g. Cohen's *d*, Pearson's *r*), indicating how they were calculated

Our web collection on statistics for biologists contains articles on many of the points above.

Software and code

Policy information about availability of computer code

Data collection	1. All experimental data of in vitro droplet assay were collected by a confocol microscope (Leica TCS SP8). The software was Leica Application Suite X. 2. All experimental data of EMSAs were collected by a scanner (Amersham Typhoon). The software was Amersham Typhoon Scanner Control Software 2.0. 3. The FRAP data was collected by a spinning-disk confocal microscopy (PerkinElmer UltraView VoX). The software was Volocity 6.3. 5. 4. The super-resolution imaging data was collected by STEDYCON STED microscopy (Abberior Instruments GmbH, Göttingen, Germany). The software was STEDYCON 7.1.53. 5. The qPCR data was collected by Tianlong Gentier 48 E Real-time PCR system. The software is built into the instrument.
Data analysis	All images, including in vitro droplet assay, EMSAs and FRAP were analyzed with Fiji (ImageJ Version: 2.0.0-rc-61/1.52n).<br>Code of STED data analysis is available in https://github.com/michaelGuo1204/FETshell_STED; Code of mathematical model and its reproduction guidance is available in https://github.com/michaelGuo1204/FETshell_PFModel;

For manuscripts utilizing custom algorithms or software that are central to the research but not yet described in published literature, software must be made available to editors and reviewers. We strongly encourage code deposition in a community repository (e.g. GitHub). See the Nature Portfolio guidelines for submitting code & software for further information.

Data

Policy information about availability of data

All manuscripts must include a data availability statement. This statement should provide the following information, where applicable:

- Accession codes, unique identifiers, or web links for publicly available datasets
- A description of any restrictions on data availability
- For clinical datasets or third party data, please ensure that the statement adheres to our policy

All data generated or analysed during this study are included in this published article (and its supplementary information files).

Research involving human participants, their data, or biological material

Policy information about studies with human participants or human data. See also policy information about sex, gender (identity/presentation), and sexual orientation and race, ethnicity and racism.

Reporting on sex and gender	Not relevant, because our work was an in vitro biophysics and biochemical study.
Reporting on race, ethnicity, or other socially relevant groupings	Not relevant, because our work was an in vitro biophysics and biochemical study.
Population characteristics	Not relevant, because our work was an in vitro biophysics and biochemical study.
Recruitment	Not relevant, because our work was an in vitro biophysics and biochemical study.
Ethics oversight	Not relevant, because our work was an in vitro biophysics and biochemical study.

Note that full information on the approval of the study protocol must also be provided in the manuscript.

Field-specific reporting

Please select the one below that is the best fit for your research. If you are not sure, read the appropriate sections before making your selection.

- [x] Life sciences
- [ ] Behavioural & social sciences
- [ ] Ecological, evolutionary & environmental sciences

For a reference copy of the document with all sections, see nature.com/documents/nr-reporting-summary-flat.pdf

Life sciences study design

All studies must disclose on these points even when the disclosure is negative.

Sample size	No statistical method was performed to predetermine sample sizes. For the high-throughput microscopy experiments, the sample size, statistical test used, and the detail of the analysis are provided in figure, extended data figure captions, and Methods. All experiments were repeated at least three times. Figures show one representative experiment.
Data exclusions	No data were excluded in this study.
Replication	All experiments were replicated successfully.
Randomization	N/A
Blinding	N/A

Reporting for specific materials, systems and methods

We require information from authors about some types of materials, experimental systems and methods used in many studies. Here, indicate whether each material, system or method listed is relevant to your study. If you are not sure if a list item applies to your research, read the appropriate section before selecting a response.

Materials & experimental systems

n/a	Involved in the study
☒	☐ Antibodies
☐	☒ Eukaryotic cell lines
☒	☐ Palaeontology and archaeology
☒	☐ Animals and other organisms
☒	☐ Clinical data
☒	☐ Dual use research of concern
☒	☐ Plants

Methods

n/a	Involved in the study
☒	☐ ChIP-seq
☒	☐ Flow cytometry
☒	☐ MRI-based neuroimaging

Eukaryotic cell lines

Policy information about cell lines and Sex and Gender in Research

Cell line source(s)	human osteosarcoma U2OS cell line
Authentication	The cell lines used have not been authenticated by us.
Mycoplasma contamination	Not contaminated.
Commonly misidentified lines (See ICLAC register)	No known misidentified lines in ICLAC version 13 database.

Plants

Seed stocks	*Report on the source of all seed stocks or other plant material used. If applicable, state the seed stock centre and catalogue number. If plant specimens were collected from the field, describe the collection location, date and sampling procedures.*
Novel plant genotypes	*Describe the methods by which all novel plant genotypes were produced. This includes those generated by transgenic approaches, gene editing, chemical/radiation-based mutagenesis and hybridization. For transgenic lines, describe the transformation method, the number of independent lines analyzed and the generation upon which experiments were performed. For gene-edited lines, describe the editor used, the endogenous sequence targeted for editing, the targeting guide RNA sequence (if applicable) and how the editor was applied.*
Authentication	*Describe any authentication procedures for each seed stock used or novel genotype generated. Describe any experiments used to assess the effect of a mutation and, where applicable, how potential secondary effects (e.g. second site T-DNA insertions, mosiacism, off-target gene editing) were examined.*
