# Supplementary material for: Deciphering the molecular mechanisms of FET fusion oncoprotein–DNA hollow co-condensates
Source: Nat Commun. 2025 Nov 7;16:9823. doi: 10.1038/s41467-025-65069-4 (PMC12594852; doi:10.1038/s41467-025-65069-4)
Supplement: Supplementary file 11 — Source data [file 41467_2025_65069_MOESM11_ESM.zip › Source_Data_20250927/Source Data Checklist.pdf]

Source Data Checklist

1. Fig. 1

(1.1) All micrograph analyses (Fig. 1a, b(i), (iii), and (iv), c(i), d, and e) were performed using ImageJ (version 2.0.0-rc-59/1.5k; open-source image processing software, http://imagej.net/Contributors).

(1.2) The Excel file located in the "Fig_1" folder contains the data used for Fig. 1b(ii) and (v). MATLAB (R2016b, version 9.1.0.441655; MathWorks; https://www.mathworks.com/products/matlab.html) was employed to import these data and generate the corresponding plots. MATLAB was run on a Windows 10 operating system (version 18362.836) under an academic license (License No. 40504596).

2. Fig. 2

(2.1) All micrograph analyses (Fig. 2a and b(i)) were performed using ImageJ (version 2.0.0-rc-59/1.5k; open-source image processing software, http://imagej.net/Contributors).

(2.2) The Excel files located in the "Fig_2" folder contain the data used for Fig. 2a(v), 2a(vii), 2b(ii), and c. MATLAB (R2016b, version 9.1.0.441655; MathWorks; https://www.mathworks.com/products/matlab.html) was employed to import these data and generate the corresponding plots. MATLAB was run on a Windows 10

operating system (version 18362.836) under an academic license (License No. 40504596).

(2.3) The Excel file “Fig_2c.xlsx” located in the “Fig_2” folder contains the data for the mean intensity of dsDNA within condensates (Fig. 2c). The box plots were generated using the “boxplot” function in MATLAB (MathWorks; https://www.mathworks.com/products/matlab.html).

3. Fig. 3

(3.1) All micrograph analyses (Fig. 3a(i), b(i), c, f(i), and g(i)) were performed using ImageJ (version 2.0.0-rc-59/1.5k; open-source image processing software, http://imagej.net/Contributors).

(3.2) The Excel files located in the “Fig_3” folder contain the data used for Fig. 3a(ii), b(ii), d, f(ii), and g(ii). MATLAB (R2016b, version 9.1.0.441655; MathWorks; https://www.mathworks.com/products/matlab.html) was employed to import these data and generate the corresponding plots. MATLAB was run on a Windows 10 operating system (version 18362.836) under an academic license (License No. 40504596).

(3.3) The Excel file “Fig_3d.xlsx” located in the “Fig_3” folder contains the data for the mean intensity of RNA inside the condensates (Fig. 3d). The Excel file “Fig_3f_ii.xlsx” and “Fig_3g_ii.xlsx” located in the “Fig_3” folder contains the data for the intensity of dsDNA with ATTO647NN (Fig. 3f(ii)) and the intensity of RNA with ATTO647NN (Fig. 3f(ii)). The box plots were generated using the “boxplot”

function in MATLAB (MathWorks; https://www.mathworks.com/products/matlab.html).

(3.4) The code used for STED data analysis in Fig. f(i) and g(i) is available at: https://github.com/michaelGuo1204/FETshell_STED .

4. Fig. 4

(4.1) Code of mathematical model and its reproduction guidance is available in https://github.com/michaelGuo1204/FETshell_PFModel .

(4.2) The Excel files located in the "Fig_4" folder contain the data used for Fig. 4b and e. MATLAB (R2016b, version 9.1.0.441655; MathWorks; https://www.mathworks.com/products/matlab.html) was employed to import these data and generate the corresponding plots. MATLAB was run on a Windows 10 operating system (version 18362.836) under an academic license (License No. 40504596).

5. Fig. 5

(5.1) All micrograph analyses (Fig. 5a, b(ii)-(iii), and c(ii)-(iii)) were performed using ImageJ (version 2.0.0-rc-59/1.5k; open-source image processing software, http://imagej.net/Contributors).

(5.2) The Excel file "Fig_5c_i_ii.xlsx" in the "Fig_5" folder contains the statistical data presented in Fig. 5c.

(5.3) The Excel file "Fig_5d(iv).xlsx" located in the "Fig_5" folder contains the data for the Cy5 intensity for SNAP-FUS-ERG (Fig. 5d(iv)). The box plots were generated using the "boxplot" function in MATLAB (MathWorks; https://www.mathworks.com/products/matlab.html).

6. Supplementary Fig. 2

(6.1) All micrograph analyses (Supplementary Fig. 2a) were performed using ImageJ (version 2.0.0-rc-59/1.5k; open-source image processing software, http://imagej.net/Contributors).

(6.2) The Excel files located in the "Supplementary Fig. 2" folder contain the data used for Supplementary Fig. 2b. MATLAB (R2016b, version 9.1.0.441655; MathWorks; https://www.mathworks.com/products/matlab.html) was employed to import these data and generate the corresponding plots. MATLAB was run on a Windows 10 operating system (version 18362.836) under an academic license (License No. 40504596). The Excel file "Supplementary_Fig_2b.xlsx" in the "Supplementary Fig. 2" folder contains the statistical data presented in Supplementary Fig. 2b.

7. Supplementary Fig. 3

(7.1) All micrograph analyses (Supplementary Fig. 3a and b(i)) were performed using ImageJ (version 2.0.0-rc-59/1.5k; open-source image processing software, http://imagej.net/Contributors).

(7.2) The Excel files located in the "Supplementary Fig. 3" folder contain the data used for Supplementary Fig. 3b(ii). MATLAB (R2016b, version 9.1.0.441655; MathWorks; https://www.mathworks.com/products/matlab.html) was employed to import these data and generate the corresponding plots. MATLAB was run on a Windows 10 operating system (version 18362.836) under an academic license (License No. 40504596).

8. Supplementary Fig. 5

(8.1) The Excel files located in the "Supplementary Fig. 5" folder contain the data used for Supplementary Fig. 5a-b. MATLAB (R2016b, version 9.1.0.441655; MathWorks; https://www.mathworks.com/products/matlab.html) was employed to import these data and generate the corresponding plots. MATLAB was run on a Windows 10 operating system (version 18362.836) under an academic license (License No. 40504596).

(8.2) The Excel file "Supplementary_Fig_5b.xlsx" located in the "Supplementary Fig. 5" folder contains the data for the Nile-red intensity (Supplementary_Fig_5b). The box plots were generated using the "boxplot" function in MATLAB (MathWorks; https://www.mathworks.com/products/matlab.html).

9. Supplementary Fig. 11

(9.1) The Excel files located in the “Supplementary Fig. 11” folder contain the data used for Supplementary Fig. 11. MATLAB (R2016b, version 9.1.0.441655; MathWorks; https://www.mathworks.com/products/matlab.html) was employed to import these data and generate the corresponding plots. MATLAB was run on a Windows 10 operating system (version 18362.836) under an academic license (License No. 40504596).
